# RAB17 promotes endometrial cancer progression by inhibiting TFRC-dependent ferroptosis

**DOI:** 10.1038/s41419-024-07013-w

**Published:** 2024-09-06

**Authors:** Xing Zhou, Miaomiao Nie, Xiaoyan Xin, Teng Hua, Jun Zhang, Rui Shi, Kejun Dong, Wan Shu, Bei Yan, Hongbo Wang

**Affiliations:** 1 Department of Obstetrics and Gynecology, Union Hospital, Tongji Medical College, Huazhong University of Science and Technology, Wuhan, Hubei 430022 P. R. China; 2 Institute of Medical Sciences, General Hospital of Ningxia Medical University, Yinchuan, Ningxia 750004 China

**Keywords:** Oncogenes, Tumour biomarkers, Nutrient signalling, Ubiquitylation

## Abstract

Studies have indicated that RAB17 expression levels are associated with tumor malignancy, and RAB17 is more highly expressed in endometrial cancer (EC) tissues than in peritumoral tissues. However, the roles and potential mechanisms of RAB17 in EC remain undefined. The present study confirmed that the expression of RAB17 facilitates EC progression by suppressing cellular ferroptosis-like alterations. Mechanistically, RAB17 attenuated ferroptosis in EC cells by inhibiting transferrin receptor (TFRC) protein expression in a ubiquitin proteasome-dependent manner. Because EC is a blood-deprived tumor with a poor energy supply, the relationship between RAB17 and hypoglycemia was investigated. RAB17 expression was increased in EC cells incubated in low-glucose medium. Moreover, low-glucose medium limited EC cell ferroptosis and promoted EC progression through the RAB17-TFRC axis. The in vitro results were corroborated by in vivo studies and clinical data. Overall, the present study revealed that increased RAB17 promotes the survival of EC cells during glucose deprivation by inhibiting the onset of TFRC-dependent ferroptosis.

## Introduction

Endometrial cancer (EC) is a common malignant tumor that occurs in the female reproductive system [1, 2]. In developed countries, the mortality rate of EC is only surpassed by that of ovarian cancer, and the incidence of EC has been increasing annually in recent years [3, 4]. There are few clinical options for treating EC, and with the advances in treatment modalities, >95% of patients with localized EC survive 5 years after diagnosis [5, 6]. However, distant metastases from EC, such as rectal and bladder metastases, are common in patients with EC. Once the tumor metastasizes or spreads to distant sites, <17% of patients with EC survive beyond 5 years. In addition, EC has low sensitivity to radiotherapy and chemotherapy, and these treatments have high toxicity and many adverse effects with poor efficacy [1, 2, 5]. The induction of ferroptosis has recently emerged as a promising therapeutic modality. Resistance to ferroptosis has been demonstrated to be a crucial mechanism in the malignant progression of EC, but its therapeutic target remains unclear.

Studies have indicated that even under aerobic conditions, cancer cells utilize energy in the form of high-rate anaerobic glycolysis, referred to as the Warburg effect, which is characterized by enhanced glucose uptake and a shift in glucose metabolism to aerobic glycolysis and the pentose phosphate pathway [7, 8]. This metabolic phenotype confers a cellular growth advantage during tumor cell proliferation by supporting the de novo synthesis of energy required for substance production and genome replication, as well as by counteracting the production of structural components of reactive oxygen species, anabolic precursors, and redox equivalents [7–9]. In addition, certain intermediate metabolites function as cofactors in signaling pathways that lead to tumor growth and proliferation [10–12].

RAB17 is the first identified epithelial cell-specific small GTPase whose expression is induced upon epithelial cell polarization [13]. Unlike other Rab proteins that are equally expressed in polarized and nonpolarized cells, RAB17 exhibits epithelial cell specificity [13, 14]. Recent studies have suggested that RAB17 is an anticancer gene that is repressed during invasive migration [15]. Previous studies have also shown that ERK2 suppresses RAB17 levels to promote breast cancer invasion [16]. Moreover, reduced levels of RAB17 have been reported to be associated with increased invasiveness of hepatocellular carcinoma [17, 18]. Collectively, these studies suggest that RAB17 may inhibit cell invasion and tumor progression. However, studies have shown that RAB17 is highly expressed in ovarian cancer cells [19]. Moreover, RAB17 knockdown increases the sensitivity of ovarian cancer cells to paclitaxel, inhibits proliferation, and leads to G1 phase arrest in the ovarian cancer cell cycle [19]. Consistent with this, our previous study also suggested a potential role for RAB17 in promoting the EC cell cycle, proliferation, and metastasis [20]. Thus, these conflicting results imply that the role of RAB17 is multifaceted, complex, and heterogeneous across cancers. Furthermore, as EC is a common malignancy, anaerobic glycolysis is also present during EC progression [21]. Moreover, some reports have proposed that the RAB family plays an important role in glucose metabolism [22, 23], but the role of RAB17 in EC is still not well defined. Therefore, the aim of the present study was to further explore the biological role of RAB17 in EC and its associated mechanisms.

## Materials and methods

### Cell culture

The Ishikawa and HEC-1A cell lines were purchased from Bena Culture Collection (Beijing, China) and cultured in high-glucose DMEM (Gibco, NY, USA) supplemented with 10% fetal bovine serum (Gibco) and 1% penicillin/streptomycin (HyClone, UT, USA). Cells were maintained at 37 °C in 5% CO_2_. For low-glucose cultures, cells were cultured in low-glucose DMEM (Gibco) supplemented with 10% fetal bovine serum (Gibco) and 1% penicillin/streptomycin (Gibco). The cell lines used in this study were not detected in the common misidentified cell line database maintained by the International Cell Line Authentication Committee and NCBI Biosample. The cell lines were verified by short tandem repeat analysis, and no mycoplasma contamination was detected.

### Transfection

The siRNAs and protein expression plasmids used in this study were constructed by RiboBio (Guangzhou, China). For transfection, siRNAs and plasmids were transfected into EC cells using Lipo3000 (Invitrogen, Carlsbad, MA, USA) according to the manufacturer’s instructions. The siRNAs were transfected at a final concentration of 50 nM, and the protein expression plasmids were transfected at a density of 2 μg with 2ul Lipo3000 reagent per 12-well plates. At 24 h after transfection, cells were used for subsequent experiments.

### Infection

The RAB17 overexpression lentivirus used in this study was produced by GeneChem (Shanghai, China). The human RAB17 gene was inserted between the AgeI and EcoRI sites of vector GV308. The recombinant lentivirus was propagated in 293 T cells and purified by centrifugation and subsequent dialysis. The viral titers were determined by fluorescence and drug screening, and the recombinant viruses were stored in a virus preservation solution at − 80 °C. Infection with lentivirus was performed according to the manufacturer’s instructions. The screening drug puromycin was added 72 h after infection, and its concentration was maintained at 5 μg/ml for 24 h. At 24 h after infection, cells were used for subsequent experiments. All the reagents utilised for the experiments described in this section were purchased from GeneChem (Shanghai, China).

### EdU staining assay

The indicated cells were fixed with 4% paraformaldehyde for 15 min and then subjected to EdU staining (Beyotime, C0071S, Wuhan, China) according to the manufacturer’s instructions. Specifically, about 1 × 10^5^ cells were seeded in 24-well plates and maintained for 24 h before the assay. A total of 250 µL EdU (10 µM) reagent was added to each well and incubated for 2 h to label the cells. After three times wash with PBS, cells were fixed in a 4% paraformaldehyde solution (Dingguo Biotechnology, AR-0211) for 15 min, permeabilized with 0.3% Triton X-100 (GenStar, VA11410) for another 15 min, and then incubated with the click-reaction reagent for 30 min at room temperature in the dark environment.Images were acquired using an inverted fluorescence microscope (Zeiss, Oberkochen, Germany), and images from at least five areas were acquired and counted for each set of assays.

### Cell Counting Kit-8

The indicated cells were seeded into 96-well plates (3×10^3^/well) for the indicated times, and medium supplemented with 10% Cell Counting Kit-8 (CCK8) reagent (Beyotime, C0037, Wuhan, China) was added to the cells, followed by incubation at 37 °C for 2 h in 5% CO_2_. The absorbance of the enzyme marker was then measured at 450 nm.

### Western blot analysis

Proteins from the indicated cells were extracted using RIPA buffer (Sigma‒Aldrich, R0278, Darmstadt, Germany) supplemented with 1% phenylmethylsulfonyl fluoride (Beyotime, ST505, Wuhan, China) and phosphatase inhibitors (Sigma‒Aldrich, P0044, Darmstadt, Germany). The protein concentration in each sample was determined with a BCA protein assay kit (Beyotime, P0009, Wuhan, China). Denatured protein samples (10 μg) were separated by 8% SDS‒PAGE (Zoman, ZD320, Beijing, China) and transferred onto PVDF membranes (Zoman, ISEQ00010, Beijing, China). The PVDF membranes were blocked with 5% nonfat dried milk (Zoman, ZS404, Beijing, China) in Tris‐buffered saline (pH 7.4) containing 0.1% Tween 20 (Zoman, ZS405-2, Beijing, China) for 1 h and subsequently incubated with specific primary antibodies in TBST at 4 °C overnight. The PVDF membranes were then washed three times in TBST for 10 min each, followed by incubation with HRP-conjugated AffiniPure goat anti-rabbit IgG (H + L) (Proteintech, SA00001-2, Wuhan, China, 1:4000) for 1 h at room temperature. Signals were detected using enhanced chemiluminescence (Meilunbio, MT0024, Dalian, China). An anti-GAPDH antibody was used to normalize protein expression. For CHX chase assays, the indicated cells were treated with 20 mg/mL CHX for 24 h after transfection and collected at the indicated time points. The cell lysates were then subjected to Western blot analysis. The following primary antibodies were used: RAB17 (Proteintech, 17501-1-AP, Wuhan, China, 1:800 dilution), TFRC (ABclonal, A5865, Wuhan, China, 1:800 dilution), ACSL4 (Proteintech, 66617-1-Ig, Wuhan, China, 1:1000 dilution), FHC (Proteintech, 10785-1-AP, Wuhan, China, 1:800 dilution), COX2 (Proteintech, 66351-1-Ig, Wuhan, China, 1:500 dilution), GPX4 (Proteintech, 67763-1-Ig, Wuhan, China, 1:1000 dilution), SLC7A11 (Proteintech, 26864-1-AP, Wuhan, China, 1:800 dilution), FLC (Abclonal, A21962, Wuhan, China, 1:500 dilution) and GAPDH (Proteintech, 60004-1-Ig, Wuhan, China, 1:5000 dilution). The original full and uncropped Western blots could be found in Supplemental Material.

### qRT‒PCR

Total RNA was isolated from tissues and cells using TRIzol (Thermo Fisher Scientific, 15596018, WI, USA). The RNA concentration and purity were measured using a spectrophotometer. RNA was reverse transcribed using a PrimeScript RT Reagent Kit (Takara, RR047Q, Dalian, China). qPCR was performed using SYBR Premix Ex Taq (Takara, RR420A, Dalian, China) according to the manufacturer’s protocol, and the expression levels were normalized to the endogenous level of GAPDH as the control. The thermocycling conditions were as follows: initial denaturation at 95 °C for 30 s; followed by 60 cycles of denaturation at 95 °C for 5 s, annealing at 55 °C for 30 s, and extension at 72 °C for 30 s. The 2^-ΔΔCT^ method was used to determine relative mRNA levels. The following primers were used: RAB17 forward, 5ʹ-TGCGCTTCTGGTGTACGAC-3ʹ; RAB17 reverse, 5ʹ-GTTCAGTTTGGCCGAAGTTTC-3ʹ; TFRC forward, 5ʹ-ACCATTGTCATATACCCGGTTCA-3ʹ; TFRC reverse, 5ʹ-CAATAGCCCAAGTAGCCAATCAT-3ʹ; GAPDH forward, 5ʹ- GGAGCGAGATCCCTCCAAAAT-3ʹ; and GAPDH reverse, 5ʹ- GGCTGTTGTCATACTTCTCATGG-3ʹ.

### Lipid peroxidation analysis

To assess the level of ferroptosis in EC cells after drug treatment, the levels of reactive oxygen species (ROS), lipid peroxidation, and mitochondrial damage were measured. Specifically, ROS levels were assessed using a Reactive Oxygen Species Assay Kit (Beyotime, S0033S, Wuhan, China) according to the manufacturer’s instructions [24]. The lipid peroxidation level was assessed using a C11-BODIPY Reagent Set (Thermo Fisher Scientific, 2115250, WI, USA) according to the manufacturer’s instructions [25]. The levels of GSH (A006-2-1), MDA (A003-1-2), and SOD (A001-3-2) were measured using commercially available kits from Nanjing Jiancheng Bioengineering Institute (Nanjing, China) according to the manufacturer’s instructions [26].

### Mitochondrial damage analysis

Mitochondrial damage was assessed using an enhanced mitochondrial membrane potential assay kit with JC-1 (Beyotime, C2003S, Wuhan, China) according to the manufacturer’s instructions [27]. In addition, EC cells were assessed by fluorescence microscopy (Zeiss, Oberkochen, Germany) to analyze the degree of ferroptosis in cells treated with the indicated drugs.

### Iron measurement

Total elemental iron was measured using a tissue iron assay kit (A039-2-1, Jiancheng, Nanjing, China) according to the manufacturer’s instructions [28]. A Phen Green SK probe was used to monitor the iron content in EC cells using a Phen Green SK Reagent Set (Thermo Fisher Scientific, 2115250, WI, USA) according to the manufacturer’s instructions [28]. The iron content of the tissue was analyzed qualitatively by Prussian blue staining of paraffin sections.

### Transmission electron microscopy

The indicated cells were freshly obtained and immediately fixed in 2.5% phosphate-glutaraldehyde for 4 h. After two washes with dimethylarsinic acid sodium buffer, the samples were directly dehydrated in an ethanol gradient, fixed, embedded, and sectioned. The samples were then viewed using a Tecnai 10 (100 kV) transmission electron microscope (FEI). For each sample, five fields of view were randomly selected, and 20 mitochondria were examined in each field of view.

### Immunofluorescence

The indicated cells that had migrated were fixed in 4% paraformaldehyde for 10 min at room temperature. The cells were then washed twice with PBS (15 min each) and permeabilized with 0.3% Triton X-100 (Beyotime, P0096, Wuhan, China) for 10 min. The cells were then incubated with blocking buffer for 30 min, followed by incubation with an anti-RAB17 primary antibody overnight. The cells were washed twice with PBS, and the nuclei were visualized using DAPI. Images were acquired using an inverted fluorescence microscope (Zeiss, Oberkochen, Germany), and images of at least five areas were acquired for each group of experiments and then analyzed. The following primary antibodies were used: RAB17 (Proteintech, 17501-1-AP, 1:50 dilution) and TFRC (ABclonal, A5865, Wuhan, China, 1:100 dilution).

### Xenograft assay

Six-week-old female BALB/c nude mice were purchased from Byrness Weil Biotechnology Ltd. (Chengdu, China), randomly divided and housed in a specific pathogen-free environment with a 12-h light/dark cycle and controlled temperature and humidity. Food and water were provided *ad libitum*. Cells (3×10^6^) were collected and injected subcutaneously into mice. At least five mice were used in each group in each experiment. The mice were then euthanized at the indicated times after injection. Each tumor was dissected, fixed with 4% formaldehyde, and embedded in paraffin. Tumor growth was monitored weekly by caliper measurements, and tumor volume was calculated using the following formula: volume=1/2 × longest diameter × (shortest diameter)^2^. All laboratory animal procedures were performed in accordance with the NIH Guide for the Care and Use of Laboratory Animals. The investigator was blinded to the group allocation of the mice during the experiment. All operations were approved by the Animal Care and Use Committee of Tongji Medical College, Huazhong University of Science and Technology.

### EC samples and IHC

A total of 204 patients with EC who underwent curative surgery between October 2015 and December 2017 at Union Hospital, Tongji Medical College, Huazhong University of Science and Technology (Wuhan, China) were included in this study. The diagnosis of EC was confirmed by the original histopathological reports. The patients were divided into two cohorts. Cohort 1 included 118 patients. Cohort 2 including 86 patients was used for analysis of the relationship between the expression levels of RAB17 and prognosis of EC patients.

Samples were paraffin-embedded, cut into sections (thickness of 5 μm), placed on slides, and stained with hematoxylin and eosin or subjected to immunohistochemical staining (IHC). Tissue sections were dewaxed and antigenically repaired using 0.01 M citrate buffer (pH 6.0) for 15 min at 95 °C, followed by incubation with primary antibody at 4 °C. After washing three times with TBS, the sections were incubated with HRP-coupled AffiniPure goat anti-rabbit IgG for 1 h at room temperature. Human EC tissue microarrays were purchased from GeneChem (Shanghai, China). The use of human EC samples and associated databases was approved by the Research Ethics Committee of Union Hospital, Tongji Medical College, Huazhong University of Science and Technology and complied with all relevant regulations. All tissue samples were collected in accordance with the informed consent policy. EC sections were stained with the indicated antibodies or nonspecific IgG antibodies as negative controls. The staining of tissue sections was scored quantitatively based on the percentage of positive cells and the intensity of staining. The following primary antibodies were used: RAB17 (Proteintech, 17501-1-AP, 1:200 dilution), TFRC (ABclonal, A5865, 1:200 dilution), and KI67 (Proteintech, 27309-1-AP, Wuhan, China, 1:1000 dilution). The following scores were assigned to sections: 0, 0% tumor cells; 1, 0–1% tumor cells; 2, 2-10% tumor cells; 3, 11-30% tumor cells; 4, 31-70% tumor cells; and 5, 71-100% tumor cells. In addition, staining intensity was rated on a scale of 0-3 as follows: 0, negative; 1, weak; 2, moderate; and 3, strong. The scale and intensity scores were then combined to obtain a total score (range, 1–8). The expression levels of indicated proteins were classified as low if the score was less than 5 and as high if the score was 5 or higher. IHC results for human tissues were scored by two independent observers.

### Coimmunoprecipitation

Coimmunoprecipitation (CoIP) assays were performed using a CoIP kit (Abs955, Absin, Shanghai, China) according to the manufacturer’s protocol. Briefly, the indicated cells were lysed with IP lysis buffer [20 mM Tris-HCl (pH 7.5), 0.5% NP-40, 250 mM NaCl, 3 mM EDTA, 3 mM EGTA, 1 mM DTT, 1 mM cocktail, 1 mM phosphoSTOP, 1 mM NEM, and 1 mm NAM]. The lysate (500 μg) was then incubated for 4 h with the indicated primary antibody or IgG as a negative control, and protein A/G-Sepharose beads were incubated with the samples for 2 h at 4 °C. After extensive washing with PBS, the immunoprecipitates were used for subsequent assays. The following antibodies were used: RAB17 (Proteintech, 17501-1-AP, 1:100 dilution) and TFRC (ABclonal, A5865, 1:100 dilution).

### Reagents and applications

MG132 (S2619) and CHX (S7418) were purchased from Selleckchem (Texas, USA). Lactacystin (HY-16594) and chloroquine (HY-17589A) were purchased from MedChemExpress (MCE, NJ, USA).

### Statistical analysis

All the statistical analyses were performed using SPSS 22.0 (IBM Corp.), and the figures were produced using GraphPad Prism 6.0 or R software. The data are expressed as the mean ± standard deviation (SD). Depending on the experiment type, the data were analyzed using an unpaired Student’s t test or one-way ANOVA followed by the Bonferroni post hoc correction where appropriate. The chi-square test was used to analyze the clinical correlation between RAB17 expression and clinicopathological features. Significant prognostic factors identified in the univariate analysis were further analyzed via multivariate analysis using Cox proportional hazards regression models. The Kaplan‒Meier method was used to estimate survival rates, and the log-rank sum test was used to assess differences between survival curves. Statistical significance was evaluated based on *P* values, and *P* < 0.05 was considered to indicate statistical significance.

## Results

### RAB17 modulates EC cell progression

**Fig. 1:**
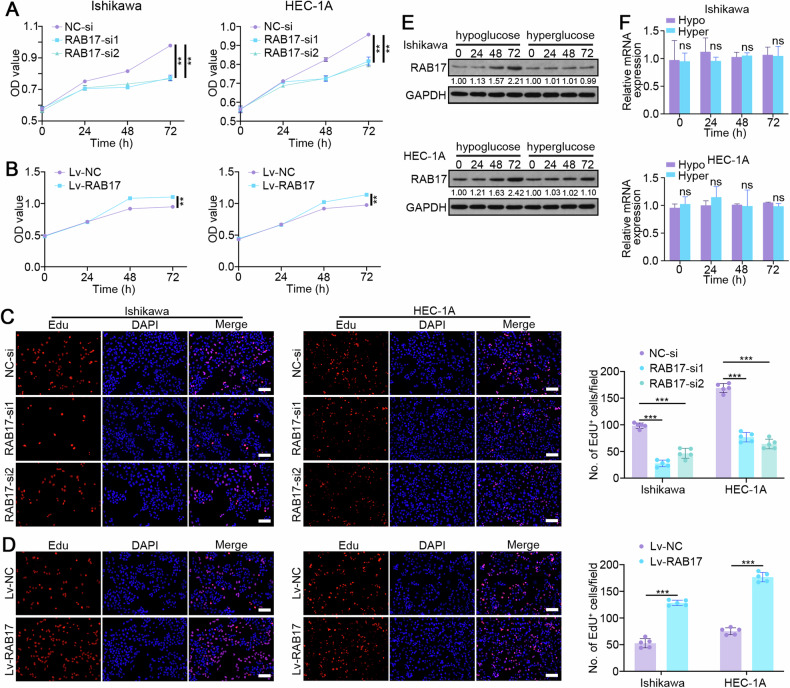
RAB17 regulates EC cell proliferation. **A** CCK-8 assays of Ishikawa and HEC-1A cell lines transfected with normal control siRNA (NC-si) or RAB17 siRNA (RAB17-si). **B** CCK-8 assays of Ishikawa and HEC-1A cells infected with normal control lentivirus (Lv-NC) or RAB17 overexpression lentivirus (Lv-RAB17). **C** Results from the EdU assays of Ishikawa and HEC-1A cells transfected with NC-si or RAB17-si for 48 h. Scale bars, 200 μm. **D** Results from the EdU assays of Ishikawa and HEC-1A cells infected with Lv-NC or Lv-RAB17 for 48 h. Scale bars, 200 μm. **E** Western blot analysis and (**F**) qRT–PCR analysis of RAB17 expression in Ishikawa and HEC-1A cells cultured in hyperglycemic (Hyper) or hypoglycemic (Hypo) medium for 72 h, respectively. GAPDH was used as an internal control. All the above assays were independently performed in triplicate (*N* = 3). The data are presented as the means ± SDs. The statistical analyses were performed by two-tailed unpaired Student’s *t* tests. **P < 0.01, and ***P < 0.001.

Our previous study indicated that RAB17 regulates EC cell progression by modulating proliferation, the cell cycle, and metastasis [20], but the underlying mechanisms remain unknown. CCK8 assays showed that RAB17 knockdown inhibited the proliferation of Ishikawa and HEC-1A cells (Fig. 1A), while RAB17 overexpression promoted the proliferation of Ishikawa and HEC-1A cells (Fig. 1B). Consistent with these findings, EdU incorporation experiments showed that RAB17 silencing markedly inhibited EC cell proliferation (Fig. 1C and Supplementary Fig. S1A), while RAB17 overexpression markedly promoted EC cell proliferation (Fig. 1D and Supplementary Fig. S1B). Because hypoglycemia is a common biological feature that promotes EC progression, the present study investigated whether RAB17 is altered under hypoglycemic conditions. Western blot analysis suggested that RAB17 protein levels were significantly elevated after incubation with low-glucose medium for 72 h (Fig. 1E). However, the qRT‒PCR analysis suggested that incubation with low-glucose medium did not significantly alter RAB17 mRNA levels (Fig. 1F), suggesting that the increase in RAB17 protein expression under hypoglycemic conditions occurred mainly at the posttranscriptional level.

### RAB17 regulates ferroptosis in EC cells

**Fig. 2:**
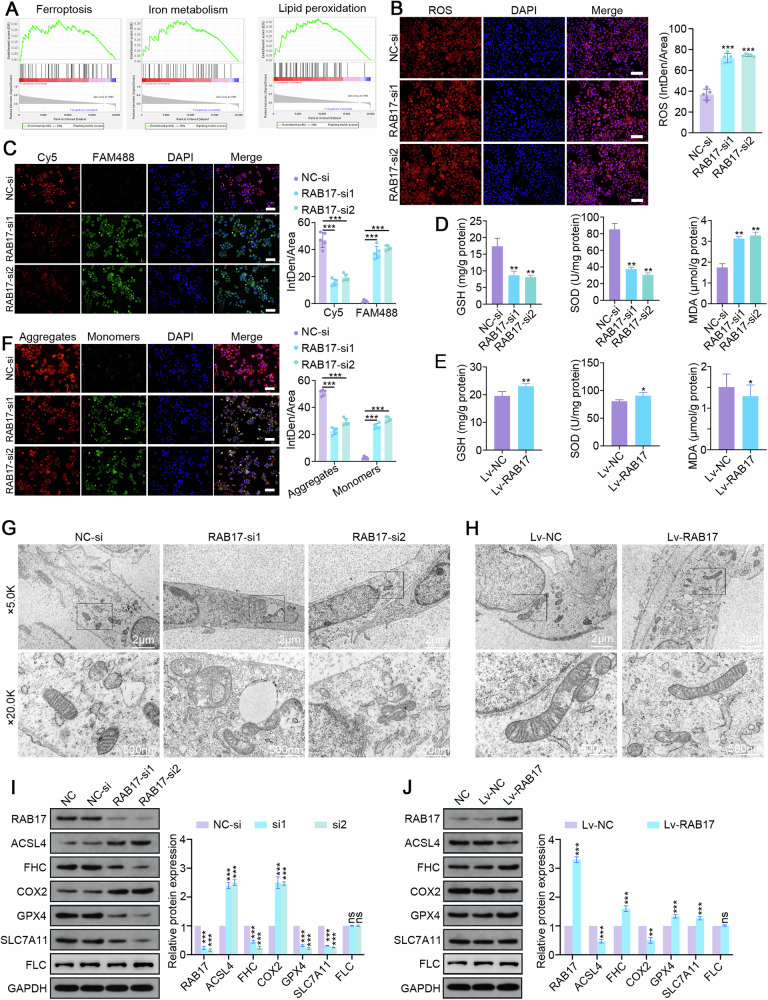
RAB17 regulates ferroptosis in EC cells. **A** Single-gene GSEA of RAB17 based on TCGA dataset. Representative images of immunofluorescence staining with (**B**) an ROS probe and (**C**) a C11-BODIPY probe in Ishikawa cell lines transfected with NC-si or RAB17-si. Scale bars, 200 μm. **D** GSH, SOD, and MDA levels in Ishikawa cell lines transfected with NC-si or RAB17-si. **E** GSH, SOD, and MDA levels in Ishikawa cell lines infected with Lv-NC or Lv-RAB17. **F** Representative images of immunofluorescence staining with a JC-1 probe in Ishikawa cell lines transfected with NC-si or RAB17-si. Scale bars, 200 μm. **G** Representative transmission electron microscopy images of Ishikawa and HEC-1A cells transfected with NC-si or RAB17-si. **H** Representative transmission electron microscopy images of Ishikawa and HEC-1A cells infected with Lv-NC or Lv-RAB17. **I** Western blot analysis of designated marker proteins for ferroptosis in Ishikawa cells transfected with NC-si or RAB17-si. **J** Western blot analysis of designated ferroptosis marker proteins in Ishikawa cells infected with Lv-NC or Lv-RAB17. GAPDH was used as an internal control. All the above assays were independently performed in triplicate (*N* = 3). The data are presented as the means ± SDs. The statistical analyses were performed by two-tailed unpaired Student’s *t* tests. *P < 0.05, **P < 0.01, and ***P < 0.001.

To further investigate how RAB17 regulates EC progression, a single-gene gene set enrichment analysis (GSEA) was performed for RAB17 using the EC dataset from TCGA database. Ferroptosis and its associated signaling pathways were the pathways most strongly associated with RAB17 expression (Fig. 2A). Reactive oxygen species (ROS) are among the critical triggers leading to ferroptosis [29, 30]. As expected, the immunofluorescence results revealed a significant increase in intracellular ROS levels in Ishikawa and HEC-1A cells after RAB17 knockdown (Fig. 2B and Supplementary Fig. S2A), but the intracellular ROS levels in Ishikawa and HEC-1A cells were decreased after RAB17 overexpression (Supplementary Fig. S2B). Detection of lipid peroxidation levels using the C11-BODIPY probe demonstrated that the lipid peroxidation levels in Ishikawa and HEC-1A cells were significantly increased after RAB17 knockdown (Fig. 2C and Supplementary Fig. S3A). In contrast, RAB17 overexpression significantly reduced the lipid peroxidation levels in Ishikawa and HEC-1A cells (Supplementary Fig. S3B). An increase in ROS promotes the susceptibility to oxidative stress damage. In Ishikawa cells, RAB17 deficiency significantly reduced the levels of the GSH and SOD antioxidant enzymes, but it significantly increased the levels of MDA (Fig. 2D). However, RAB17 overexpression in Ishikawa cells significantly increased the intracellular GSH and SOD levels but decreased the MDA levels (Fig. 2E). Mitochondrial damage is one of the characteristic alterations of ferroptosis [30, 31]. JC-1 staining indicated that mitochondrial damage was significantly increased in Ishikawa and HEC-1A cells after RAB17 knockdown (Fig. 2F and Supplementary Fig. S4A), while RAB17 overexpression significantly decreased intracellular mitochondrial damage in Ishikawa and HEC-1A cells (Supplementary Fig. S4B). To visualize the alterations occurring in the cellular substructure, cells were evaluated by transmission electron microscopy. RAB17 knockdown decreased cellular mitochondrial size, increased membrane density, decreased mitochondrial ridges, and disrupted the outer mitochondrial membrane (Fig. 2G). In contrast, RAB17 overexpression attenuated the mitochondrial structural alterations in EC cells, as indicated by an intact outer mitochondrial membrane, smooth mitochondria, and intact mitochondria with slight swelling (Fig. 2H). Because altered intracellular iron content is one of the major manifestations of ferroptosis [30–32], P-GSK staining was performed to analyze the iron content. The iron content was significantly increased in Ishikawa and HEC-1A cells with low RAB17 expression (Supplementary Fig. S5A), but it was significantly decreased in Ishikawa and HEC-1A cells with high RAB17 expression (Supplementary Fig. S5B). Consistent results were obtained for the relative quantification of iron levels (Supplementary Fig. S5C). Finally, the alteration of ferroptosis markers in EC cells with different RAB17 levels was verified by Western blot analysis. As expected, the levels of the ACSL4 and COX2 ferroptosis markers were significantly increased in Ishikawa and HEC-1A cells after RAB17 knockdown, while the levels of the FHC, GPX4, and SLC7A11 ferroptosis inhibitors were significantly decreased after RAB17 knockdown (Fig. 2I and Supplementary Fig. S5D). Conversely, RAB17 overexpression significantly decreased the levels of ACSL4 and COX2 but increased the levels of FHC, GPX4 and SLC7A11 in Ishikawa and HEC-1A cells (Fig. 2J and Supplementary Fig. S5E). Notably, the aberrant of RAB17 expression did not affect the protein levels of FLC, as displayed by the Western blotting of Fig. 2I, J. Collectively, these results suggested that RAB17 affects EC progression by regulating ferroptosis.

### RAB17 represses TFRC expression at the posttranscriptional level

**Fig. 3:**
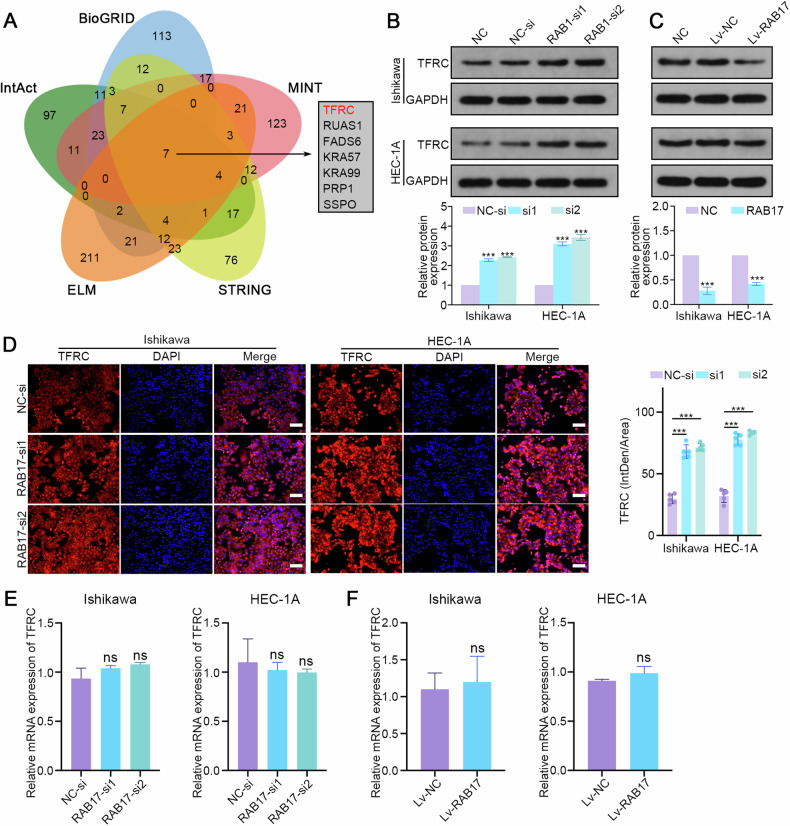
RAB17 regulates TFRC expression in EC cells at the posttranscriptional level. **A** RAB17-binding proteins predicted based on the BioGRID, IntAct, MINT, ELM, and STRING databases. **B** Western blot analysis of TFRC expression in Ishikawa and HEC-1A cells transfected with NC-si or RAB17-si. **C** Western blot analysis of TFRC expression in Ishikawa and HEC-1A cells infected with Lv-NC or Lv-RAB17. **D** Representative images of IF staining showing TFRC expression in Ishikawa and HEC-1A cells transfected with NC-si or RAB17-si. Scale bars, 200 μm. **E** qRT–PCR analysis of TFRC expression in Ishikawa and HEC-1A cells transfected with NC-si or RAB17-si. **F** qRT–PCR analysis of TFRC expression in Ishikawa and HEC-1A cells infected with Lv-NC or Lv-RAB17. GAPDH was used as an internal control. All the above assays were independently performed in triplicate (*N* = 3). The data are presented as the means ± SDs. The statistical analyses were performed by two-tailed unpaired Student’s *t* tests. ***P < 0.001.

To further explore the direct targets of RAB17 in regulating ferroptosis, multiple protein interaction databases (BioGRID, IntAct, MINT, ELM, and STRING) [33–37] were used to predict the proteins that interact with RAB17. The integrated analysis suggested that TFRC, a cell surface receptor for cellular iron uptake through endocytosis, was a potential direct target of RAB17 (Fig. 3A). Western blot analysis suggested that TFRC protein levels were significantly increased after RAB17 knockdown (Fig. 3B) but significantly decreased after RAB17 overexpression in Ishikawa and HEC-1A cells (Fig. 3C). Immunofluorescence verified the above results. Specifically, the fluorescence intensity of TFRC was significantly enhanced in Ishikawa and HEC-1A cells with RAB17 knockdown (Fig. 3D), whereas the fluorescence intensity of TFRC was significantly decreased when RAB17 was overexpressed in Ishikawa and HEC-1A cells (Supplementary Fig. S6A). The TFRC mRNA levels were quantified using qRT‒PCR to determine whether RAB17 affects TFRC zprotein levels by regulating TFRC mRNA. However, altered RAB17 expression did not affect the TFRC mRNA level (Fig. 3E, F), suggesting that RAB17 regulates TFRC expression at the posttranscriptional level.

### RAB17 promotes TFRC degradation via the NEDD4L-dependent ubiquitin‒proteasome pathway in ECs

**Fig. 4:**
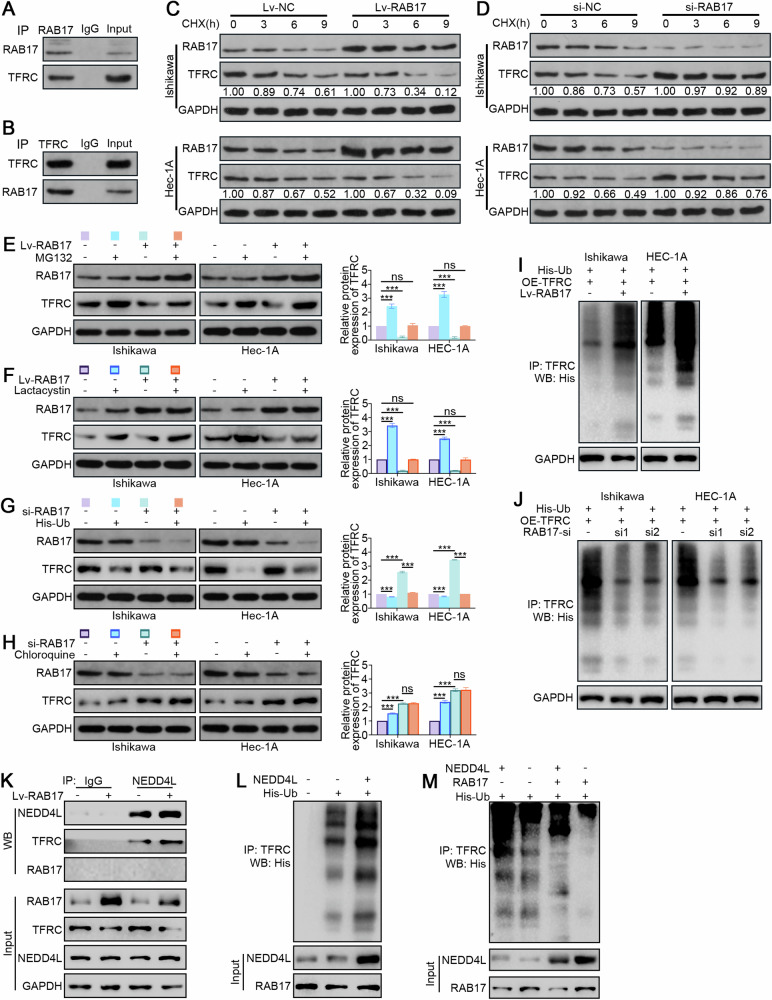
RAB17 regulates TFRC expression in EC cells through the ubiquitin-proteasome pathway. **A** Ishikawa cell lysates were incubated with an anti-RAF17 antibody, and interacting proteins were detected by Western blot analysis with an anti-TFRC antibody. **B** Ishikawa cell lysates were incubated with an anti-TFRC antibody, and interacting proteins were detected by Western blot analysis with an anti-RAB17 antibody. **C** Ishikawa and HEC-1A cells were infected with Lv-NC and Lv-RAB17. CHX (20 μmol) was added for the indicated time, and the cell lysates were subjected to Western blot analysis for RAB17 and TFRC. **D** Ishikawa and HEC-1A cells were transfected with NC-si or RAB17-si1. CHX (20 μmol) was added for the indicated time, and the cell lysates were subjected to Western blot analysis for RAB17 and TFRC. **E** Ishikawa and HEC-1A cells were infected with Lv-NC and Lv-RAB17. The cells were then treated with the MG132 proteasome inhibitor (20 mmol) for 12 h, and Western blot analysis was performed with anti-RAB17 and anti-TFRC antibodies. **F** Ishikawa and HEC-1A cells were transfected with Lv-NC or Lv-RAB17. The cells were then treated with the lactacystin proteasome inhibitor (10 mmol) for 12 h, and Western blot analysis was performed with anti-RAB17 and anti-TFRC antibodies. **G** Ishikawa and HEC-1A cells were transfected with NC-si or RAB17-si1. The cells were then transfected with His-tagged ubiquitin-containing vectors (His-Ub) for 12 h, and Western blot analysis was performed with anti-RAB17 and anti-TFRC antibodies. **H** Ishikawa and HEC-1A cells were transfected with NC-si or RAB17-si1. The cells were then treated with the chloroquine lysosomal inhibitor (10 mmol) for 12 h, and Western blot analysis was performed with anti-RAB17 and anti-TFRC antibodies. **I**, **J** Ishikawa and HEC-1A cells were transfected as indicated and treated with MG132 for 12 h. Lysates were immunoprecipitated with anti-TFRC and detected with anti-His. GAPDH was used as an internal control. **K** Ishikawa cells were infected with Lv-CTL or Lv-RAB17, and cell lysates were immunoprecipitated with the indicated primary antibody and immunoblotted as indicated. **L**, **M** Ishikawa cells were transfected as indicated, and then cell lysates were immunoprecipitated with anti-TFRC antibody and detected with anti-His antibody. All the above assays were independently performed in triplicate (*N* = 3). ***P < 0.001.

The mechanism by which RAB17 regulates TFRC was investigated by immunoprecipitation assays to determine whether there is a direct association between the RAB17 and TFRC proteins. A RAB17 antibody was used to immunoprecipitate Ishikawa cell lysates, and Western blot analysis indicated direct binding between TFRC and RAB17 (Fig. 4A). Similarly, immunoprecipitation using a TFRC antibody, followed by Western blot analysis, further verified the direct binding between RAB17 and TFRC (Fig. 4B). To demonstrate that RAB17 posttranscriptionally regulates TFRC protein levels, Ishikawa and HEC-1A infected with Lv-RAB17 and control (Lv-NC) lentiviruses were treated with CHX. Western blot analysis indicated that RAB17 overexpression significantly promoted TFRC degradation (Fig. 4C). CHX treatment of Ishikawa and HEC-1A cells transfected with RAB17-si and control siRNA suggested that the degradation of TFRC was inhibited after RAB17 knockdown (Fig. 4D). To further explore the potential regulatory mechanism of RAB17 on TFRC, Ishikawa and HEC-1A cells were treated with MG132 (proteasome inhibitor) and chloroquine (lysosome inhibitor). Western blot analysis demonstrated that MG132 significantly inhibited the regulatory effect of RAB17 on TFRC (Fig. 4E), and this affect was verified using lactacystin, another proteasome inhibitor (Fig. 4F). Moreover, His-Ub suppressed the increase in intracellular TFRC after RAB17-si treatment (Fig. 4G), while chloroquine did not affect this increase (Fig. 4H), suggesting that the regulation of TFRC by RAB17 may occur through the ubiquitin‒proteasome pathway. Finally, to demonstrate that RAB17 promotes the degradation of ubiquitinated TFRC, Ishikawa and HEC-1A cells were transfected with the His-Ub plasmid and infected with the Lv-RAB17 virus (Fig. 4I). Western blot analysis indicated that exogenous overexpression of RAB17 significantly promoted the degradation of ubiquitinated TFRC relative to that of Lv-NC. However, the ubiquitination-mediated degradation of TFRC was inhibited by silencing RAB17 expression (Fig. 4J).

E3 ligases are critical factors of the degradation of ubiquitinated proteins [38]. Since RAB17 does not contain domains identified as motifs for ubiquitin binding, we hypothesized that RAB17 regulates TFRC ubiquitination by affecting the binding of TFRC and E3 ligases. Notably, studies have proposed that NEDD4L is the E3 ligase mediating TFRC ubiquitination [39]. The CoIP analysis further showed that the binding of NEDD4L to TFRC was markedly enhanced when RAB17 was overexpressed, whereas RAB17 and NEDD4L failed to directly bind to each other (Fig. 4K). We therefore hypothesized that RAB17 promotes NEDD4L bound to TFRC. Indeed, NEDD4L overexpression substantially increased the ubiquitylation level of the TFRC protein (Fig. 4L), whereas this increase was rescued by RAB17 silencing regardless of the presence of NEDD4L (Fig. 4M). Taken together, these data indicated that RAB17 promotes the degradation of TFRC in ECs via the NEDD4L-dependent ubiquitin‒proteasome pathway.

### RAB17 limits EC ferroptosis by facilitating TFRC degradation in low-glucose conditions

**Fig. 5:**
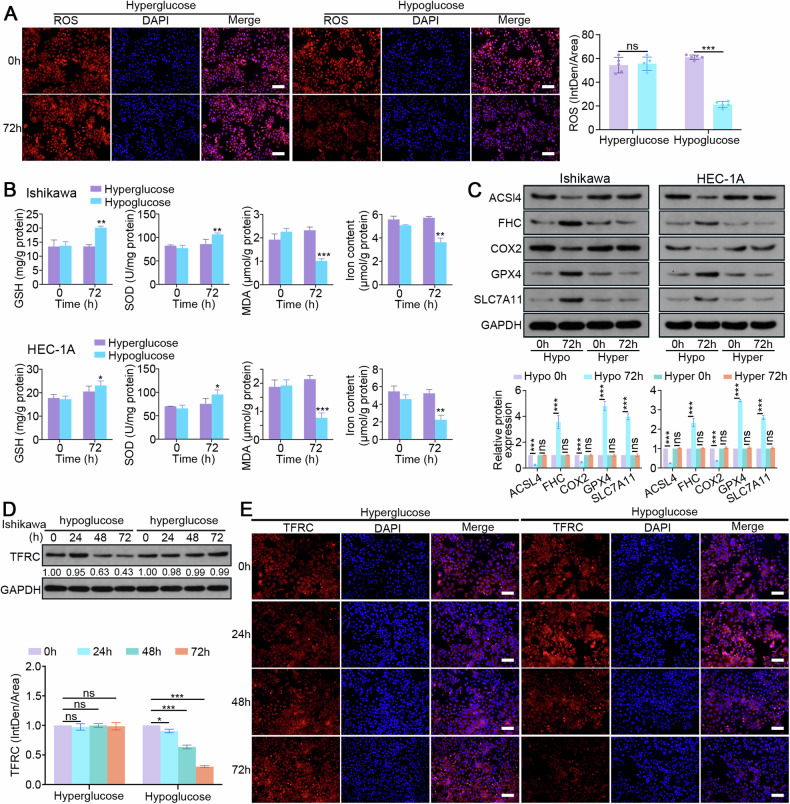
RAB17 mediates TFRC-dependent ferroptosis in a hypoglycemic state. **A** Representative images of immunofluorescence staining with an ROS probe in Ishikawa cell lines cultured in hyperglycemic (Hyper) or hypoglycemic (Hypo) medium for the designated times. Scale bars, 200 μm. **B** GSH, SOD, MDA, and levels in Ishikawa and HEC-1A cells cultured in hyperglycemic (Hyper) or hypoglycemic (Hypo) medium for the designated times. **C** Western blot analysis of designated marker proteins for ferroptosis in Ishikawa and HEC-1A cells cultured in hyperglycemic (Hyper) or hypoglycemic (Hypo) medium for the designated times. **D** Western blot analysis of TFRC expression in Ishikawa cells cultured in hyperglycemic (Hyper) or hypoglycemic (Hypo) medium for the designated times. **E** Representative images of immunofluorescence staining for TFRC in Ishikawa cell lines cultured in hyperglycemic (Hyper) or hypoglycemic (Hypo) medium for the designated times. Scale bars, 200 μm. GAPDH was used as an internal control. All the above assays were independently performed in triplicate (*N* = 3). The data are presented as the means ± SDs. The statistical analyses were performed by two-tailed unpaired Student’s *t* tests. *P < 0.05, **P < 0.01, and ***P < 0.001.

Because hypoglycemia increased RAB17 expression in EC cells (Fig. 1E), the role of a low-glucose in EC ferroptosis and tumor progression by regulating the RAB17-TFRC axis was further investigated by treating EC cells with low-glucose or high-glucose medium for 72 h. EC cells cultured in low-glucose medium exhibited decreased ROS fluorescence intensity (Fig. 5A) but increased GSH and SOD levels (Fig. 5B); however, this effect was not observed in high-glucose conditions (Fig. 5A, B). In addition, both lipid oxidation (MDA) levels and the intracellular iron content were significantly decreased (Fig. 5B). After culturing Ishikawa and HEC-1A cells in low-glucose medium for 72 h, western blot analysis demonstrated that the protein levels of ACSL4 and COX2 were significantly decreased, while the levels of FHC, GPX4 and SLC7A11 were increased (Fig. 5C). Furthermore, Ishikawa cells cultured in low-glucose medium for 72 h had significantly reduced TFRC levels, as indicated by Western blot and immunofluorescence analyses (Fig. 5D, E). However, culturing cells with either high- or low-glucose medium did not significantly affect the TFRC mRNA levels (Supplementary Fig. S6B). These results suggested that RAB17 inhibits EC ferroptosis by downregulating TFRC, which promotes tumor progression in hypoglycemic states.

### TFRC-mediated ferroptosis is essential for RAB17-mediated regulation of EC progression

**Fig. 6:**
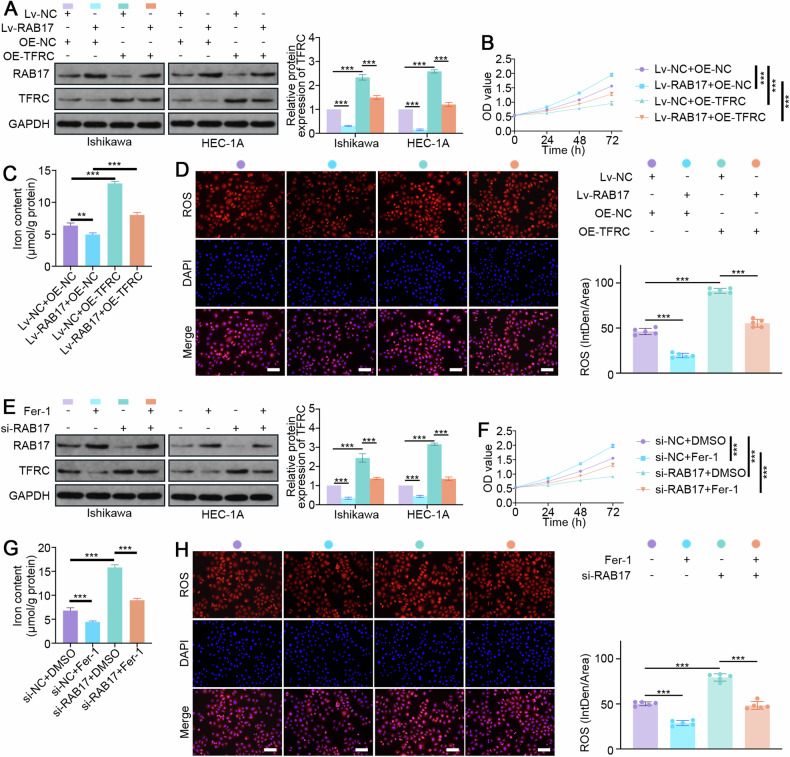
TFRC-mediated ferroptosis is critical for RAB17-mediated regulation of EC cell proliferation. **A** Western blot analysis of RAB17 and TFRC expression in Ishikawa and HEC-1A cells cotransfected with the designated vectors. **B** CCK-8 assays of Ishikawa cell lines cotransfected with the designated vectors. **C** The iron contents of Ishikawa cell lines cotransfected with the designated vectors. **D** Representative images of immunofluorescence staining with an ROS probe in Ishikawa cell lines cotransfected with the designated vectors. Scale bars, 200 μm. **E** Western blot analysis of RAB17 and TFRC expression in Ishikawa and HEC-1A cells transfected with/without designated siRNAs or treated with/without Fer-1. **F** CCK-8 assays of Ishikawa cell lines transfected with/without designated siRNAs or treated with/without Fer-1. **G** Iron content of Ishikawa cell lines transfected with/without designated siRNAs or treated with/without Fer-1. **H** Representative images of immunofluorescence staining using an ROS probe in Ishikawa cell lines transfected with/without the indicated siRNAs or treated with/without Fer-1. Scale bars, 200 μm. GAPDH was used as an internal control. All the above assays were independently performed in triplicate (*N* = 3). The data are presented as the means ± SDs. The statistical analyses were performed by two-tailed unpaired Student’s *t* tests. **P < 0.01, and ***P < 0.001.

To further demonstrate that TFRC is critical for RAB17-mediated regulation of EC progression (Fig. 6A), Ishikawa and HEC-1A cells were infected with the Lv-RAB17 lentivirus and transfected with the TFRC overexpression (TFRC-OE) plasmid. As expected, TFRC overexpression significantly inhibited the increase in cell growth promoted by RAB17 overexpression (Fig. 6B and Supplementary Fig. S7A). Relative quantitative analysis of iron content also demonstrated that TFRC overexpression significantly enhanced the decrease in iron content caused by RAB17 overexpression (Fig. 6C and Supplementary Fig. S7B). Moreover, the immunofluorescence results suggested that TFRC overexpression significantly inhibited the decrease in ROS levels caused by RAB17 overexpression (Fig. 6D and Supplementary Fig. S7C). To further verify that TFRC-mediated ferroptosis is critical for the RAB17-mediated regulation of EC progression, cells were transfected with RAB17-si to knockdown RAB17 and then treated with Fer-1 (iron death inhibitor) and DMSO. RAB17-si increased the TFRC protein level, but Fer-1 treatment significantly decreased the TFRC protein level (Fig. 6E). Similarly, the proliferation of RAB17-deficient EC cells was significantly greater after Fer-1 treatment than after DMSO treatment (Fig. 6F and Supplementary Fig. S7D). Moreover, Fer-1 treatment of RAB17-deficient EC cells significantly reduced the iron content but significantly decreased the ROS levels (Fig. 6G, H and Supplementary Fig. S7E-F). Taken together, these results suggested that TFRC-mediated ferroptosis is essential for RAB17-mediated regulation of EC progression.

### Increased RAB17 promotes EC progression in vivo by inhibiting ferroptosis

**Fig. 7:**
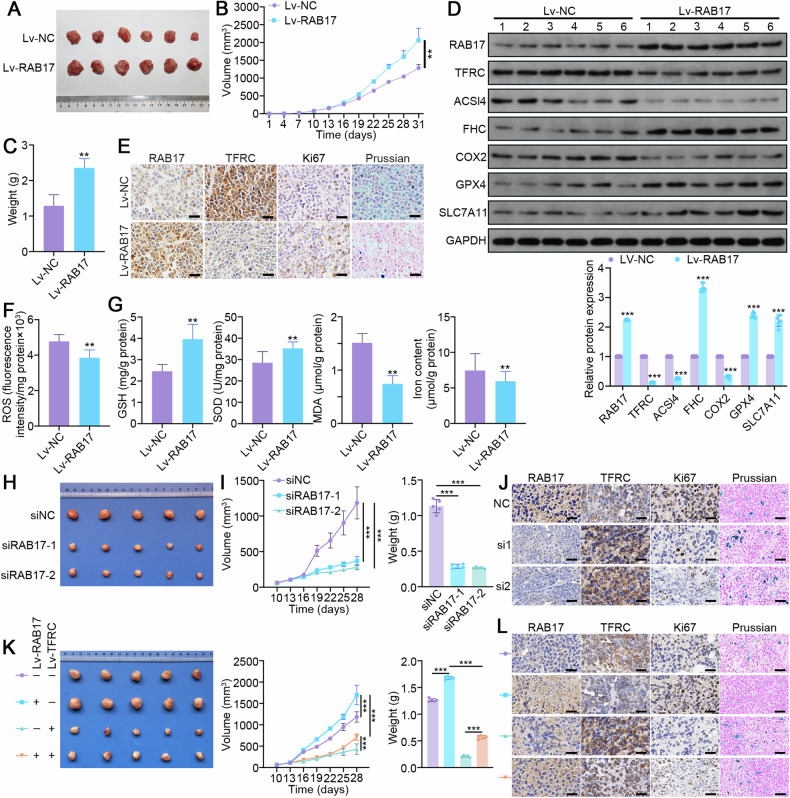
RAB17 regulates ferroptosis in ECs in vivo. **A** Representative images of the xenografts, (**B**) tumor volume, and (**C**) tumor weight 31 days after inoculation of Ishikawa cells infected with Lv-NC or Lv-RAB17 (*n* = 6 per group). The tumor volumes were measured every 3 days. **D** Western blot analysis of RAB17 and TFRC expression in Lv-NC and Lv-RAB17 xenografts. **E** Representative images of immunohistochemical staining for RAB17, TFRC, and KI67, as well as Prussian blue staining, of Lv-NC and Lv-RAB17 xenografts. Scale bars, 100 μm. **F** ROS levels in Lv-NC and Lv-RAB17 xenografts. **G** GSH, SOD, MDA, and iron levels in Lv-NC and Lv-RAB17 xenografts. **H** Western blot analysis of designated ferroptosis marker proteins in Lv-NC and Lv-RAB17 xenografts. GAPDH was used as an internal control. **H** Representative images of the xenografts, (**I**) tumor volume, and weight 28 days after inoculation of Ishikawa cells infected with siNC or siRAB17 (*n* = 5 per group). The tumor volumes were measured every 3 days. **J** Representative images of immunohistochemical staining for RAB17, TFRC, and KI67, as well as Prussian blue staining, of siNC or siRAB17 xenografts. Scale bars, 100 μm. **K** Representative images of the xenografts, tumor volume, and weight 28 days after inoculation of Ishikawa cells infected with indicated vectors (*n* = 5 per group). The tumor volumes were measured every 3 days. **L** Representative images of immunohistochemical staining for RAB17, TFRC, and KI67, as well as Prussian blue staining, of indicated xenografts. Scale bars, 100 μm. All the above assays were independently performed in triplicate (*N* = 3). The data are presented as the means ± SDs. The statistical analyses were performed by two-tailed unpaired Student’s *t* tests. **P < 0.01, and ***P < 0.001.

To further investigate whether RAB17 is involved in regulating EC progression in vivo, nude mice were subcutaneously inoculated with Ishikawa cells stably infected with Lv-RAB17 and Lv-NC (Fig. 7A). RAB17 overexpression significantly promoted tumor progression, as evidenced by accelerated tumor growth and a significant increase in tumor weight (Fig. 7A–C). Western blot and immunohistochemical analyses demonstrated that TFRC levels were significantly reduced in RAB17-overexpressing EC xenograft tumors (Fig. 7D, E). Immunohistochemical analysis also demonstrated that the Ki67 levels were significantly higher in RAB17-overexpressing EC xenograft tumors than in control tumors (Fig. 7E). As expected, the ROS levels were significantly reduced in RAB17-overexpressing EC tumors (Fig. 7F). RAB17 overexpression significantly increased the, GSH and SOD levels but significantly decreased the MDA levels and iron levels in EC tumors (Fig. 7G). In addition, Prussian blue staining was performed to qualitatively measure the tumor iron levels, which that the intratumor iron levels were significantly lower in the high RAB17 expression group compared to the control group (Fig. 7E). Western blot analysis further demonstrated that the levels of ACSL4 and COX2 ferroptosis markers were significantly lower but that the levels of FHC, GPX4 and SLC7A11 were higher in the RAB17-overexpressing EC tumors compared to the control EC tumors (Fig. 7D).

Besides, Ishikawa cells stably infected with lentivirus carrying siRAB17 or siNC were subcutaneously injected into nude mice. As expected, RAB17 knockdown significantly inhibited tumor progression (Fig. 7H, I). Immunohistochemistry analyses showed that TFRC levels were significantly increased in RAB17-deficient EC xenograft tumors (Fig. 7J). In addition, Ki67 levels were markedly lower in EC xenograft tumors with low RAB17 expression than in control tumors (Fig. 7J). Furthermore, RAB17-deficient EC tumors had significantly increased iron levels, as evidenced by the prussian blue staining (Fig. 7J).

Finally, the importance of TFRC in RAB17-mediated EC progression was investigated by infecting Ishikawa cells expressing Lv-RAB17 or Lv-NC with Lv-TFRC or Lv-CTL (Fig. 7K). The RAB17-induced tumor growth was significantly inhibited by TFRC expression (Fig. 7K). The immunohistochemistry results indicated that TFRC expression significantly promoted the onset of ferroptosis (Fig. 7L). Collectively, these results indicated that RAB17 promotes EC progression in vivo by inhibiting TFRC-dependent ferroptosis.

### High RAB17 expression is associated with reduced TFRC levels and poor prognosis in patients with EC

**Fig. 8:**
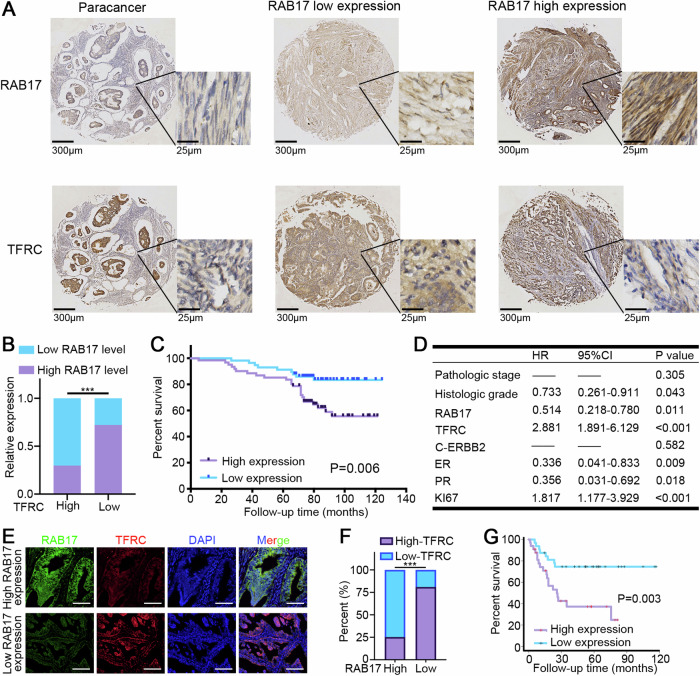
RAB17 and TFRC expression levels are significantly associated with prognosis in patients with EC. **A** Representative IHC images of RAB17 and TFRC expression in 118 EC tissues. The right panels (scale bars = 25 μm) show magnified views of the boxed area in the corresponding left panels (scale bars = 300 μm). **B** Chi-square test based on the immunohistochemical analysis of RAB17 and TFRC expression. **C** The overall survival (OS) of EC patients with different RAB17 protein expression levels were assessed by Kaplan–Meier survival curves and log-rank tests. **D** Multivariate analysis of factors associated with overall survival in patients with EC. **E** Representative immunofluorescence images of RAB17 and TFRC expression in 86 EC tissues from our cohort. **F** Correlation analysis between RAB17 and TFRC expression, chi-square test was used. **G** The overall survival (OS) of 86 EC patients from our cohort with different RAB17 protein expression levels were assessed by Kaplan–Meier survival curves and log-rank tests. ***P < 0.001.

To assess the correlation between RAB17 and TFRC expression levels in EC tissues, IHC assays were performed on 118 EC samples. High RAB17 expression was associated with reduced TFRC expression (Fig. 8A, B). Moreover, RAB17 expression was strongly correlated with tumor stage, histological grade, time of last pregnancy, erb-b2 receptor tyrosine kinase 2 (ERBB2) expression, estrogen receptor (ER) levels, progesterone receptor (PR) levels, and the Ki67 index in patients with EC (Supplementary Table S1). Univariate analysis revealed that RAB17 expression, TFRC expression, pathological stage, histological grade, ERBB2 expression, ER level, PR level, and Ki67 index were unfavorable prognostic factors for overall survival in patients with EC (Supplementary Table S2). K‒M survival analysis confirmed that OS was significantly worse in the high RAB17 expression group than in the low RAB17 expression group (Fig. 8C). Multivariate analysis further showed that RAB17 was an independent prognostic factor for overall survival in EC patients (Fig. 8D). Consistently, immunofluorescence analysis of 86 EC samples from our center suggested that RAB17 was significantly negatively correlated with TFRC expression (Fig. 8E, F). Indeed, high RAB17 expression was dramatically associated with poor EC prognosis (Fig. 8G). In short, these findings suggested that high RAB17 expression is associated with reduced TFRC levels and poor prognosis in EC patients.

## Discussion

The expression and role of RAB17 have been extensively characterized in a variety of cancer tissues [15–20]; however, the expression and function of RAB17 in EC are unknown. Our previous study revealed that RAB17 is overexpressed in EC but expressed at lower levels in adjacent paraneoplastic tissues [20]. In contrast, Wang et al. reported a complete lack of RAB17 expression in hepatocellular carcinoma [18]. The present comprehensive analysis suggested that this difference in RAB17 expression among various tumors may be associated with the tissue of origin. RAB17 is expressed in polarized and nonpolarized epithelial cells, but RAB17 expression is absent in nonepithelial cells [13, 14]. Notably, both EC and ovarian cancer originate from epithelial cells, while both hepatocellular carcinoma and breast cancer originate from nonepithelial cells. In addition, reduced RAB17 promotes tumor progression by activating the ERK2 pathway in hepatocellular carcinoma and breast cancer [16, 17]. In the present study, ERK2 was not significantly activated after RAB17 knockdown in ECs (data not shown); therefore, ERK2 and its downstream signaling was not further explored in ECs. Overall, these results imply that the role of RAB17 is heterogeneous and complex in various tumors.

The present study suggested that RAB17 expression promoted EC progression, but RAB17 did not regulate EC progression through ERK2. Therefore, a single-gene GSEA was performed, which suggested that ferroptosis-related pathways were enriched following altered RAB17 expression. The present study showed that altered RAB17 expression significantly regulated oxidative stress levels, and transmission electron microscopy revealed that mitochondrial RAB17-deficient EC cells exhibited ferroptosis-like changes. Further investigation of whether RAB17 regulates ferroptosis-related proteins or pathways demonstrated that RAB17 inhibited the production of ROS and suppressed the expression of ferroptosis-related proteins, which increased antioxidant damage capacity, reduced mitochondrial damage, and decreased ferroptosis susceptibility in EC. Overall, these results suggested that RAB17 promotes the progression of EC by inhibiting ferroptosis.

Because EC is a relatively nutrient-deprived tumor with a low energy supply [40, 41], the role of RAB17 in EC cells was investigated in hypoglycemic conditions. Under hypoglycemic conditions, RAB17 protein expression increased, which further promoted the proliferation of Ishikawa and HEC-1A cells. Thus, these results suggested that RAB17 may act as an effector in response to tumor-depleted nutrition to further promote the survival of EC cells in a hypoglycemic environment. Although the present study demonstrated that low-glucose conditions do not affect RAB17 mRNA expression levels, the exact mechanism regulating the protein expression of RAB17 under low-glucose conditions was not elucidated, indicating the need for additional studies.

Because RAB17 downregulation promoted ferroptosis-related changes in EC cells, the possible downstream targets involved in this process were investigated. Antioxidant capacity and altered iron metabolic signaling are key pathways in tumor cells that counter ferroptosis damage. Therefore, the potential of TFRC as a downstream target of RAB17 was investigated and verified by predictive analysis using online databases and further experimental evaluation. The protein levels but not the mRNA levels of TFRC were reduced after RAB17 was overexpressed, and there was a significant reduction in TFRC in EC cells cultured in low-glucose medium. Thus, the present results suggested that the overexpression of RAB17 in a low-glucose environment inhibits downstream TFRC-dependent ferroptosis to promote the survival of EC cells under energy-deficient conditions. Moreover, the present study confirmed that RAB17 directly binds to TFRC and promotes its NEDD4L-dependent ubiquitin‒proteasome degradation. These findings provide new insights into the mechanism of downstream regulation by RAB17.

The present study had several limitations. The present study only investigated ferroptosis, which is the type of cell death most significantly correlated with RAB17, but the roles of other forms of cell death, such as apoptosis and necrosis, in mediating RAB17-induced EC remain to be explored. In addition, our data only suggested that the RAB17 protein is regulated by the low-glycemic microenvironment, but the exact mechanism was not identified, which will be a promising direction for further investigation. Nevertheless, the present study suggested that RAB17 is upregulated in a hypoglycemic environment to promote EC progression by inhibiting TFRC-dependent ferroptosis. The present findings provide a foundation for a therapeutic strategy targeting the RAB17-TFRC axis in patients with EC.

## Supplementary information

Supplementary Information

Original full and uncropped Western blots

## Data Availability

The published article includes all the datasets generated and analyzed for this study.
