# Supplementary material for: RAB17 promotes endometrial cancer progression by inhibiting TFRC-dependent ferroptosis
Source: Cell Death Dis. 2024 Sep 6;15(9):655. doi: 10.1038/s41419-024-07013-w (PMC11379720; doi:10.1038/s41419-024-07013-w)
Supplement: Supplementary file 1 — Supplementary Information [file 41419_2024_7013_MOESM1_ESM.docx]

**Supplementary Information**

**RAB17 promotes endometrial cancer progression by inhibiting TFRC-dependent ferroptosis**

Xing Zhou^1,*^, Miaomiao Nie^1,*^, Xiaoyan Xin^1^, Teng Hua^1^, Jun Zhang^1^, Rui Shi^1^, Kejun Dong^1^, Wan Shu^1^, Bei Yan^2^, Hongbo Wang^1^

**Table of contents**

**Fig. S1-S7**

**Table S1-S2**

**
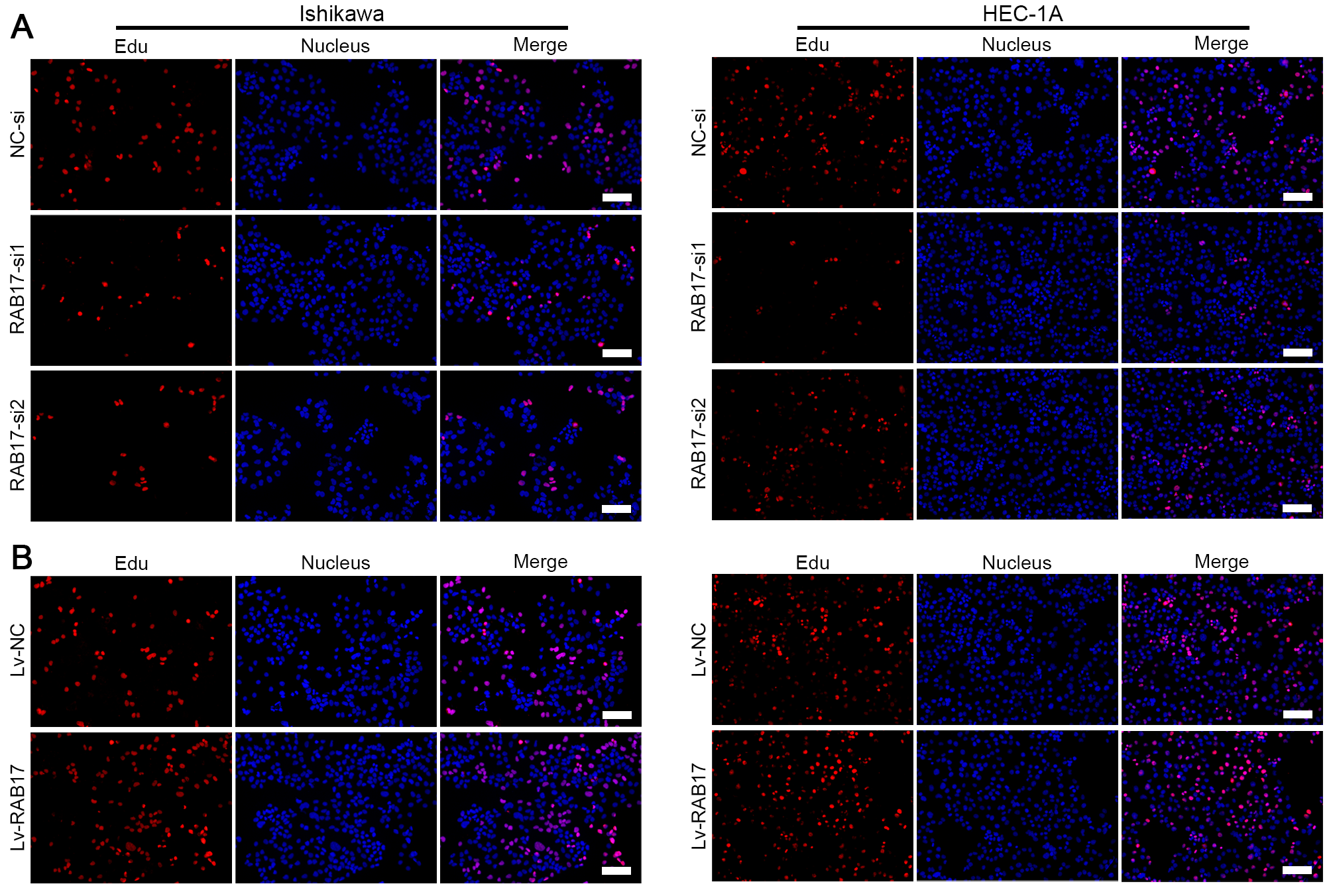
Fig. S1 RAB17 regulates cell proliferation of Ishikawa and HEC-1A.** (A) EdU assays of Ishikawa and HEC-1A cell lines transfected with NC-si or RAB17-si for 72h. Scale bars, 200 μm. (B) EdU assays of Ishikawa and HEC-1A cell lines infected with Lv-NC and Lv-RAB17 for 72h. Scale bars, 200 μm. All the above assays were independently performed in triplicate.

**
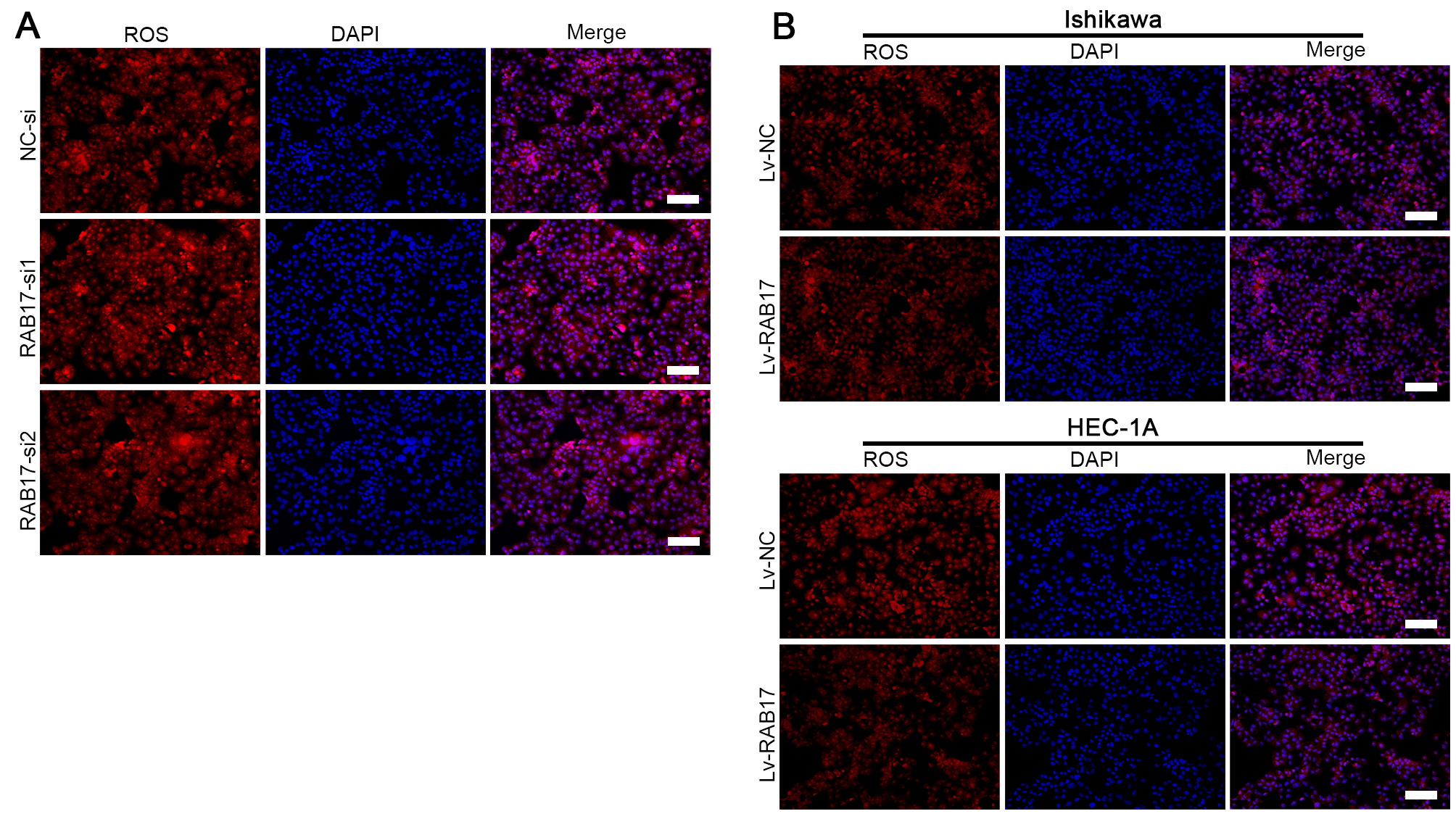
Fig. S2 RAB17 regulates ROS levels of Ishikawa and HEC-1A.** (A) Representative images of immunofluorescence staining with ROS probe in HEC-1A cell lines transfected with NC-si or RAB17-si. Scale bars, 200 μm. (B) Representative images of immunofluorescence staining with ROS probe in Ishikawa and HEC-1A cell lines infected with Lv-NC and Lv-RAB17. Scale bars, 200 μm. All the above assays were independently performed in triplicate.


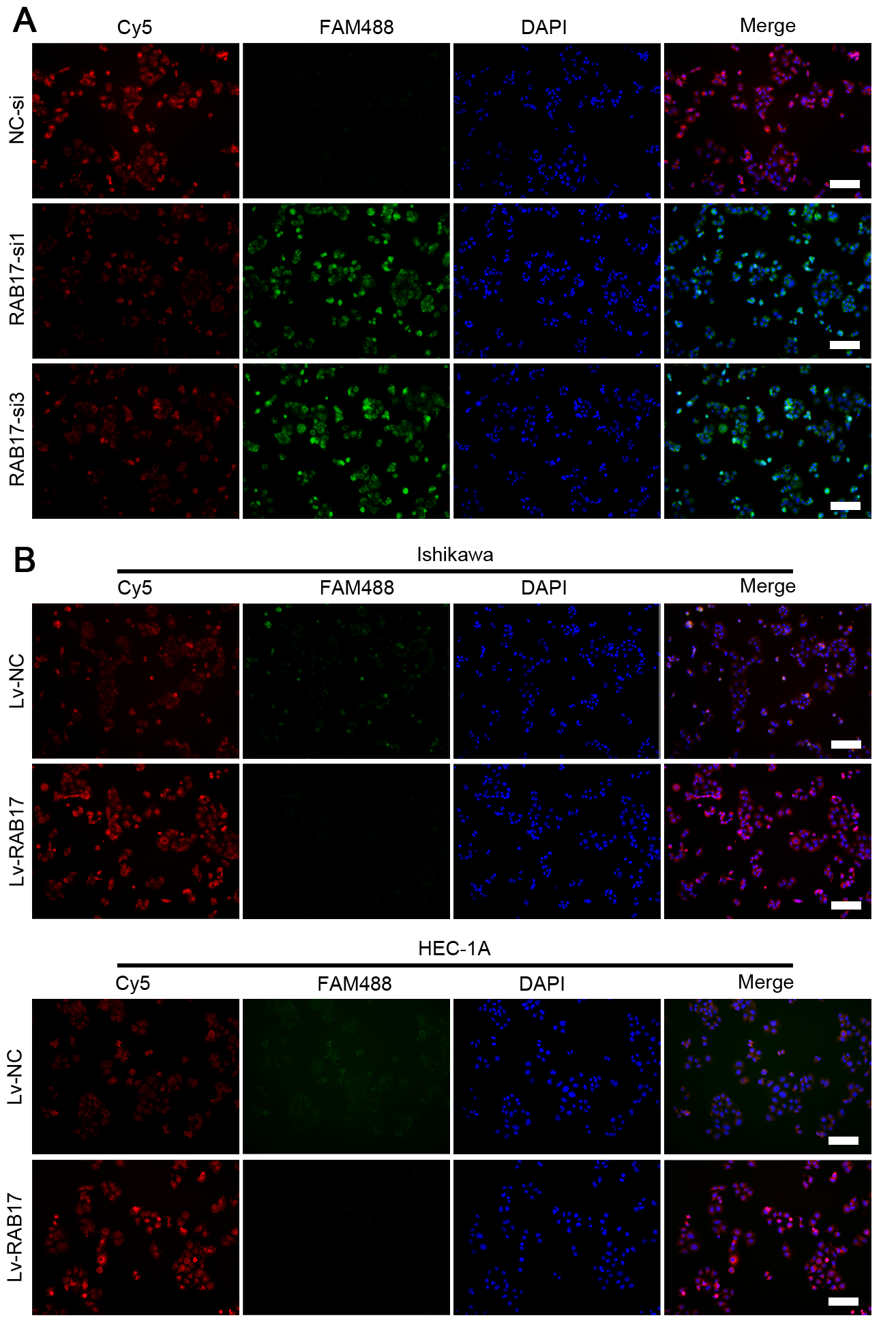


**Fig. S3 RAB17 regulates lipid peroxidation levels of Ishikawa and HEC-1A.** (A) Representative images of immunofluorescence staining with C11-BODIPY probe in HEC-1A cell lines transfected with NC-si or RAB17-si. Scale bars, 200 μm. (B) Representative images of immunofluorescence staining with C11-BODIPY probe in Ishikawa and HEC-1A cell lines infected with Lv-NC and Lv-RAB17. Scale bars, 200 μm. All the above assays were independently performed in triplicate


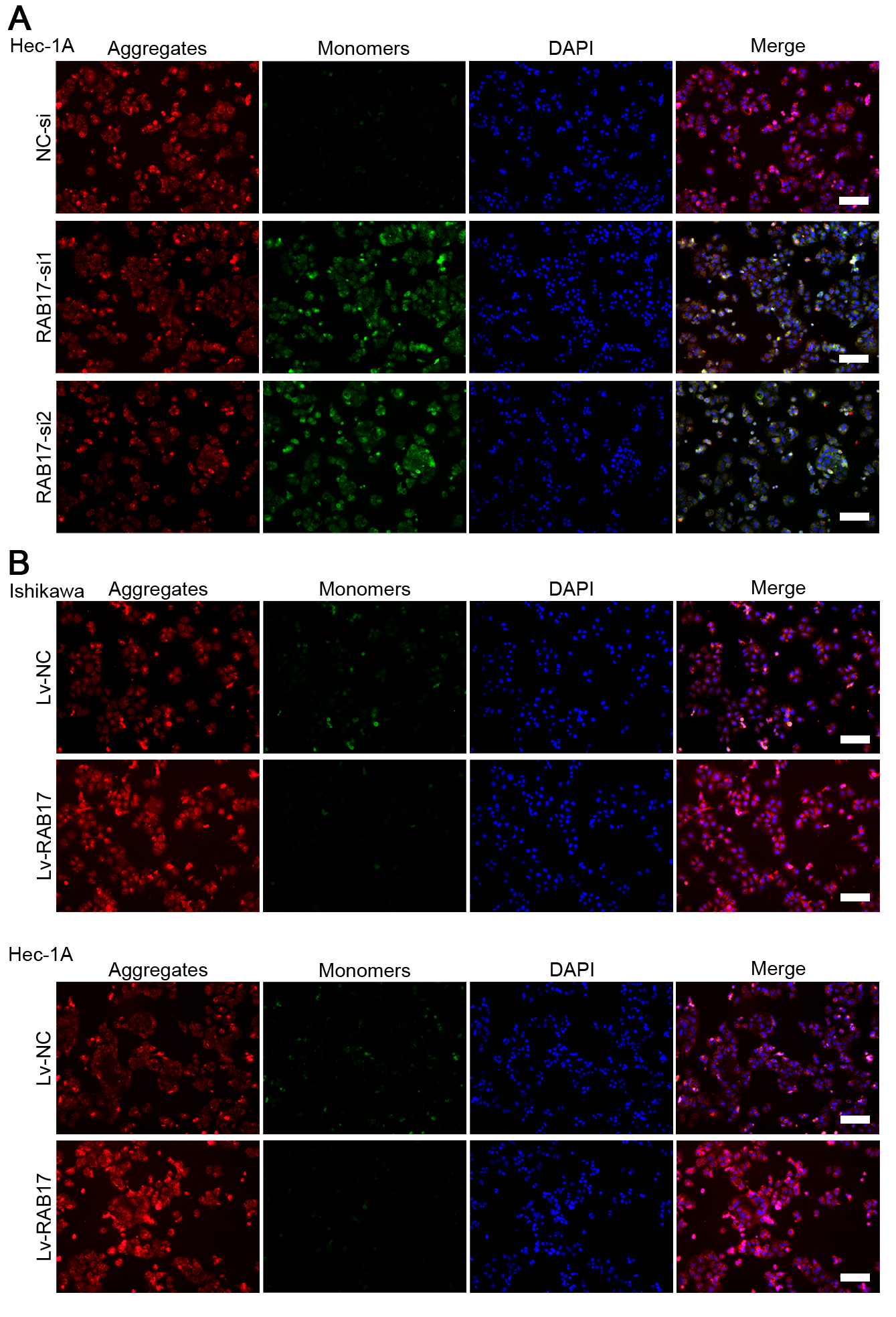


**Fig. S4 RAB17 regulates mitochondrial damage of Ishikawa and HEC-1A.** (A) Representative images of immunofluorescence staining with JC-1 probe in HEC-1A cell lines transfected with NC-si or RAB17-si. Scale bars, 200 μm. (B) Representative images of immunofluorescence staining with JC-1 probe in Ishikawa and HEC-1A cell lines infected with Lv-NC and Lv-RAB17. Scale bars, 200 μm. All the above assays were independently performed in triplicate.


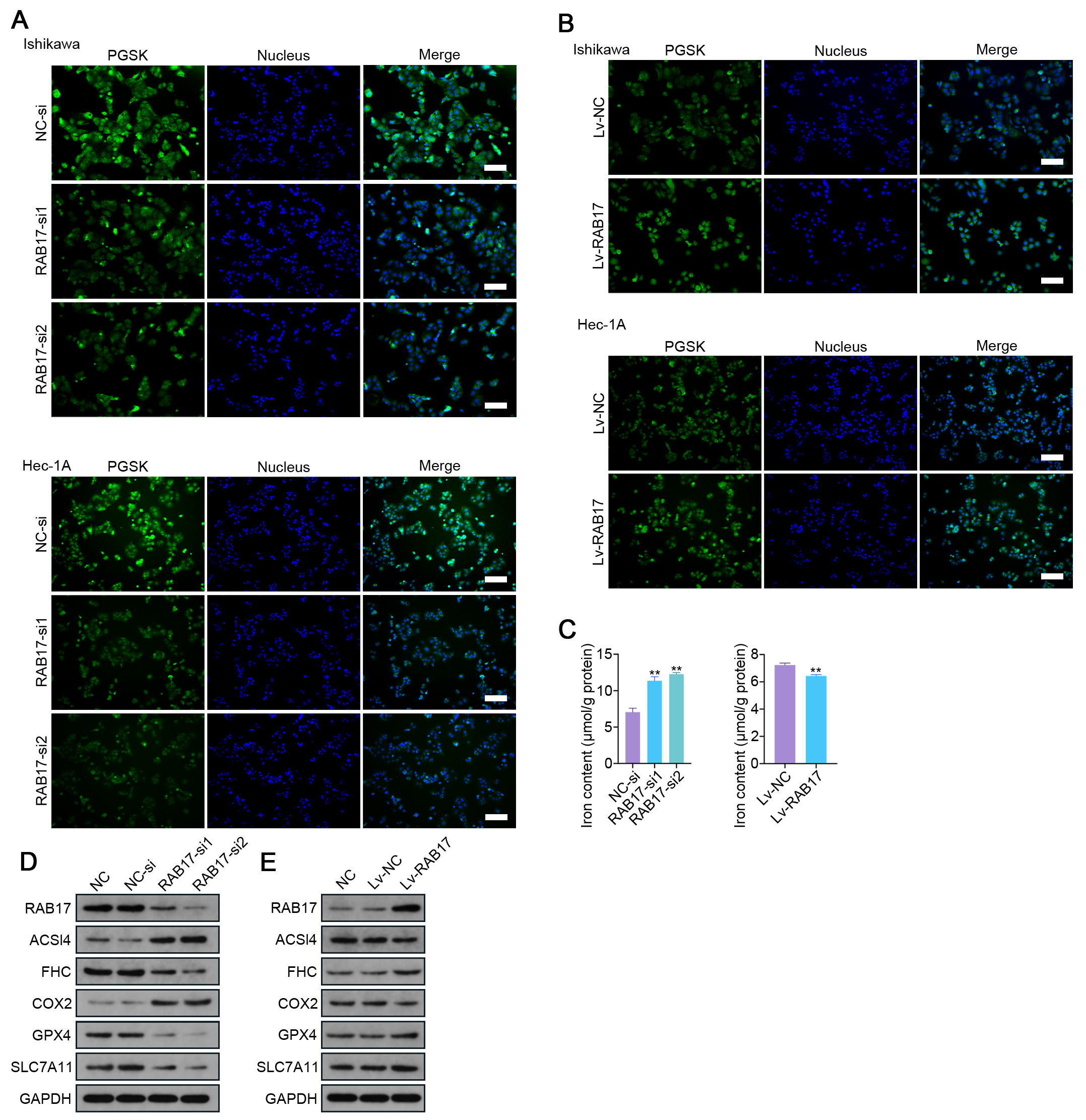


**Fig. S5 RAB17 regulates iron contents of Ishikawa and HEC-1A.** (A) Representative images of immunofluorescence staining with PGSK probe in Ishikawa and HEC-1A cell lines transfected with NC-si or RAB17-si. Scale bars, 200 μm. (B) Representative images of immunofluorescence staining with PGSK probe in Ishikawa and HEC-1A cell lines infected with Lv-NC and Lv-RAB17. Scale bars, 200 μm. All the above assays were independently performed in triplicate. (C) The iron contents of Ishikawa cell lines transfected with NC-si and RAB17-si or infected with Lv-NC and Lv-RAB17, respectively. (D) Western blot analysis of designated marker proteins for ferroptosis in HEC-1A cells transfected with NC-si or RAB17-si. (E) Western blot analysis of designated marker proteins for ferroptosis in HEC-1A cells infected with Lv-NC and Lv-RAB17. GAPDH were used as internal controls. All the above assays were independently performed in triplicate. The data are presented as the means ± SDs. The statistical analyses were performed by two-tailed unpaired Student’s t-test.

**
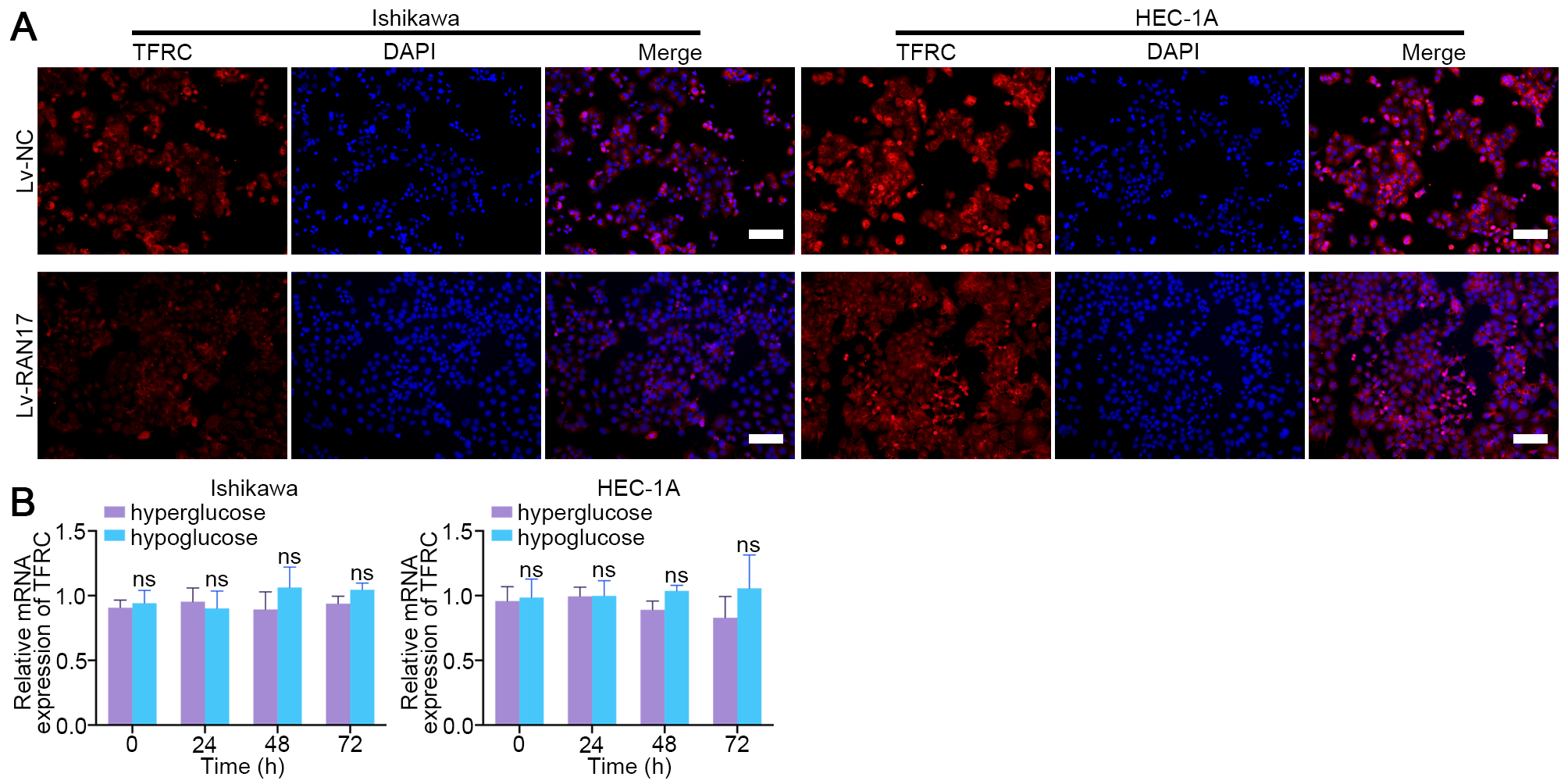
**

**Fig. S6 RAB17 regulates TFRC protein levels of Ishikawa and HEC-1A.** (A) Representative images of immunofluorescence staining with TFRC expression in Ishikawa and HEC-1A cell lines infected with Lv-NC and Lv-RAB17. Scale bars, 200 μm. (B) qRT–PCR analysis of TFRC expression in Ishikawa and HEC-1A cells cultured in hyperglycemic (Hyper) or hypoglycemic (Hypo) medium for designated time, respectively. GAPDH were used as internal controls. All the above assays were independently performed in triplicate. The data are presented as the means ± SDs. The statistical analyses were performed by two-tailed unpaired Student’s t-test.


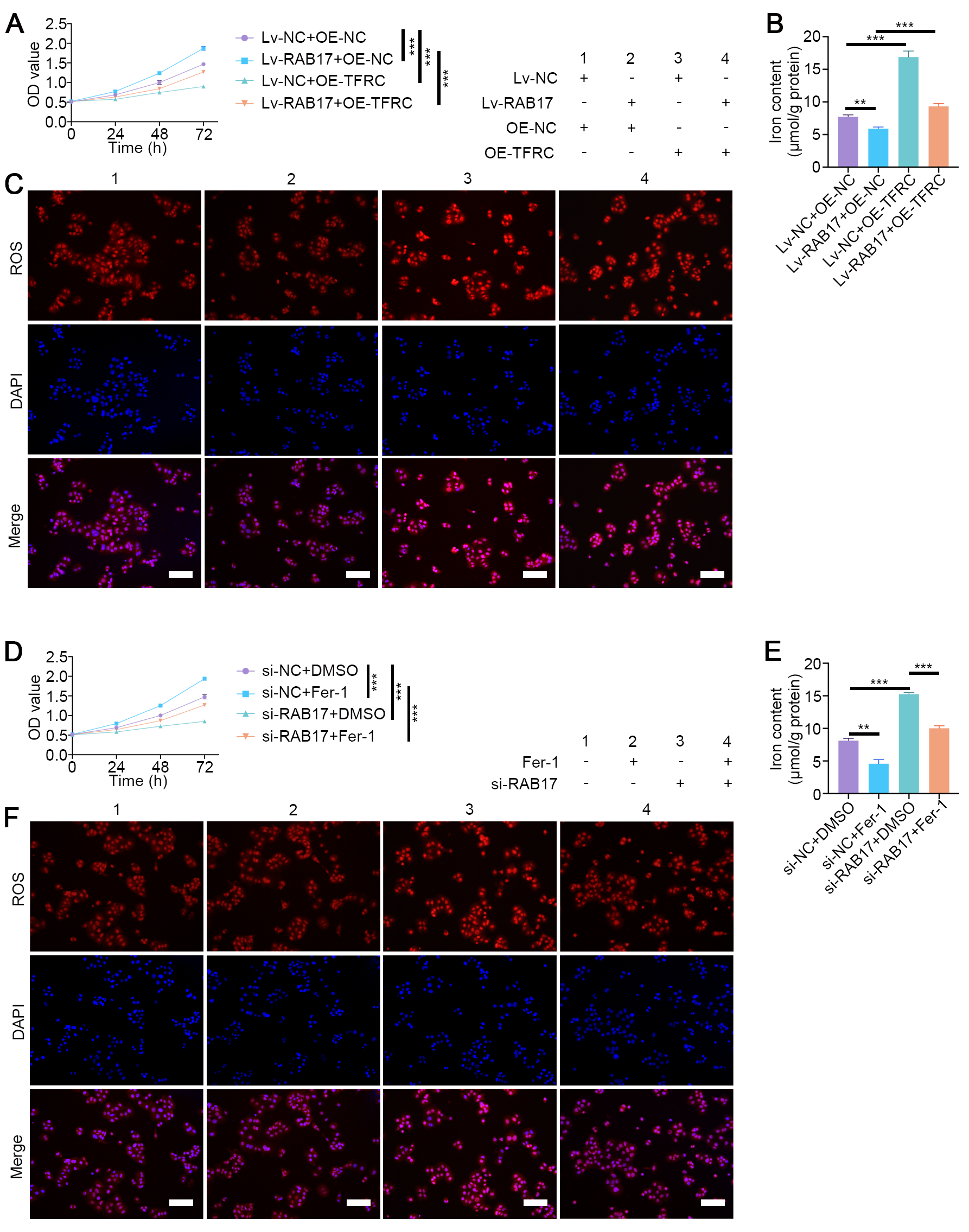


**Fig. S7 TFRC-mediated ferroptosis is critical for RAB17-regulated EC cell proliferation.** (A) CCK-8 assays of HEC-1A cell lines cotransfected with designated vectors. (B) The iron contents of HEC-1A cell lines cotransfected with designated vectors. (C) Representative images of immunofluorescence staining with ROS probe in HEC-1A cell lines cotransfected with designated vectors. Scale bars, 200 μm. (D) CCK-8 assays of HEC-1A cell lines transfected with/without designated siRNAs or treated with/without Fer-1. (E) The iron contents of HEC-1A cell lines transfected with/without designated siRNAs or treated with/without Fer-1. (F) Representative images of immunofluorescence staining with ROS probe in HEC-1A cell lines transfected with/without designated siRNAs or treated with/without Fer-1. Scale bars, 200 μm. GAPDH were used as internal controls. All the above assays were independently performed in triplicate. The data are presented as the means ± SDs. The statistical analyses were performed by two-tailed unpaired Student’s t-test.

**Supplementary Table S1: Correlation of RAB17 expression with clinicopathological parameters in endometrial cancer.**

| **Clinical features** | **N** | **IRF-1** |  | **P value** |
| --- | --- | --- | --- | --- |
|  |  | High expression | Low expression |  |
| **Age (years)** |  |  |  |  |
| <=60 | 90 | 43 | 47 | 0.837 |
| >60 | 28 | 14 | 14 |  |
| **Pathologic stage** |  |  |  |  |
| Stage1 | 61 | 10 | 51 | <0.001 |
| Stage2+3 | 57 | 47 | 10 |  |
| **Tumor diameter (cm)** |  |  |  |  |
| <=5.5 |  | 36 | 49 | 0.639 |
| >5.5 |  | 11 | 12 |  |
| **Histologic grade** |  |  |  |  |
| G3 | 45 | 15 | 30 | 0.007 |
| G1+G2 | 64 | 38 | 26 |  |
| **Time of first menstruation** |  |  |  |  |
| <=15 | 39 | 19 | 20 | 0.897 |
| >15 | 72 | 36 | 36 |  |
| **Menopausal time** |  |  |  |  |
| <=45 | 13 | 8 | 5 | 0.487 |
| >45 | 57 | 29 | 28 |  |
| **Marrying age** |  |  |  |  |
| <=22 | 50 | 23 | 27 | 0.613 |
| >22 | 63 | 32 | 31 |  |
| **Number of pregnancies** |  |  |  |  |
| <=3 | 69 | 37 | 32 | 0.170 |
| >3 | 49 | 20 | 29 |  |
| **Number of births** |  |  |  |  |
| <=3 | 88 | 43 | 45 | 0.782 |
| >3 | 25 | 13 | 12 |  |
| **Number of miscarriages** |  |  |  |  |
| 0 | 49 | 25 | 24 | 0.780 |
| >0 | 60 | 29 | 31 |  |
| **Time of first pregnancy** |  |  |  |  |
| <=25 | 80 | 40 | 40 | 0.720 |
| >25 | 24 | 11 | 13 |  |
| **Time of last pregnancy** |  |  |  |  |
| <=30 |  | 37 | 28 | 0.034 |
| >30 |  | 13 | 24 |  |
| **TFRC** |  |  |  |  |
| High expression | 61 | 17 | 44 | <0.001 |
| Low expression | 57 | 40 | 17 |  |
| **P53 mutations** |  |  |  |  |
| Negative | 44 | 23 | 21 | 0.898 |
| Positive | 41 | 22 | 19 |  |
| **C-ERBB2** |  |  |  |  |
| Negative | 60 | 24 | 36 | 0.005 |
| Positive | 18 | 14 | 4 |  |
| **ER** |  |  |  |  |
| Negative | 23 | 5 | 18 | <0.001 |
| Positive | 63 | 40 | 23 |  |
| **PR** |  |  |  |  |
| Negative | 15 | 3 | 12 | 0.005 |
| Positive | 72 | 43 | 29 |  |
| **KI67** |  |  |  |  |
| <=30% | 37 | 11 | 26 | <0.001 |
| >30% | 42 | 29 | 13 |  |

**Table S2: Univariate analysis of factors associated with survival in endometrial cancer.**

|  | HR | 95%CI | P value |
| --- | --- | --- | --- |
| Pathologic stage | 1.495 | 1.221-3.412 | 0.014 |
| Histologic grade | 1.582 | 1.146-3.372 | <0.001 |
| RAB17 expression | 3.178 | 2.334-6.399 | <0.001 |
| TFRC expression | 0.344 | 0.104-0.972 | <0.001 |
| C-ERBB2 | 0.571 | 0.161-0.951 | 0.001 |
| ER | 0.371 | 0.135-0.922 | 0.021 |
| PR | 0.406 | 0.123-0.852 | 0.016 |
| KI67 index | 0.271 | 0.008-0.712 | <0.001 |
