# Supplementary material for: RAB17 promotes endometrial cancer progression by inhibiting TFRC-dependent ferroptosis
Source: Cell Death Dis. 2024 Sep 6;15(9):655. doi: 10.1038/s41419-024-07013-w (PMC11379720; doi:10.1038/s41419-024-07013-w)
Supplement: Supplementary file 2 — Original full and uncropped Western blots [file 41419_2024_7013_MOESM2_ESM.pdf]

Figure 1E

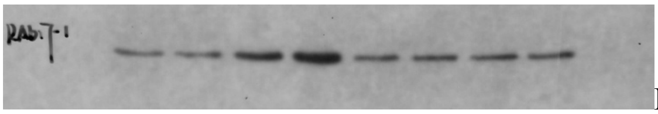
RAB17

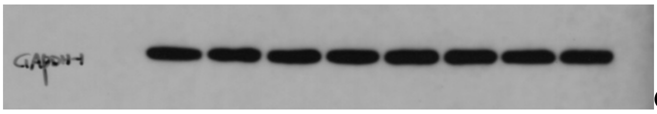
GAPDH

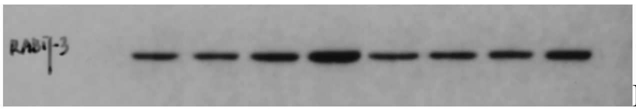
RAB17

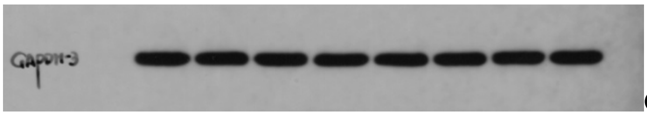
GAPDH

Figure 2I

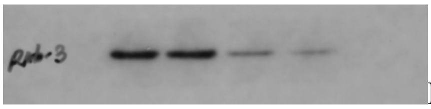
RAB17

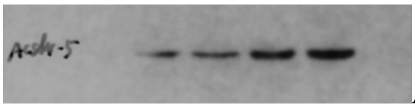
ACSL4

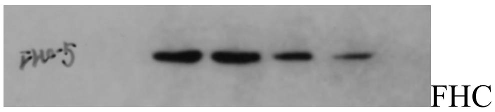
FHC

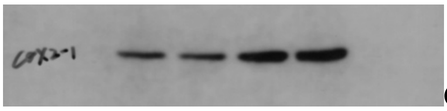
COX2

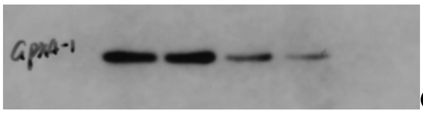
GPX4

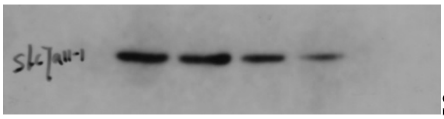
SLC7A11

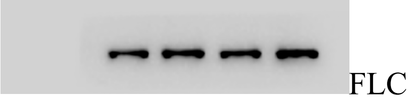
FLC

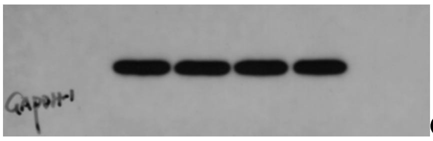
GAPDH

Figure 2J

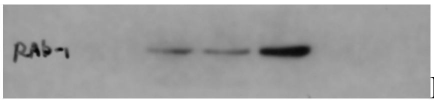
RAB17

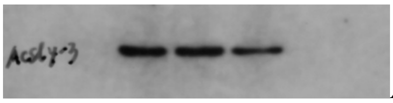
ACSL4

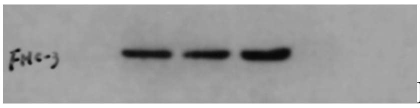

FHC

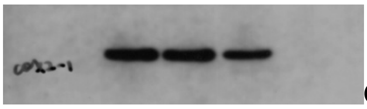

COX2

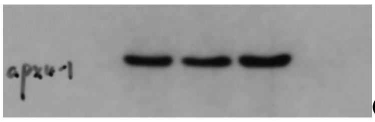

GPX4

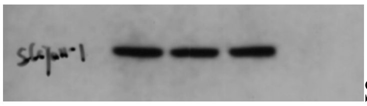

SLC7A11

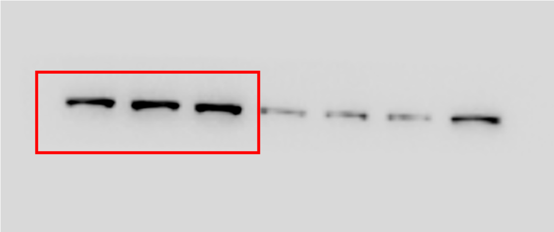

FLC

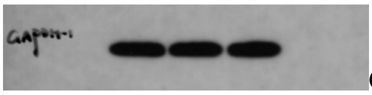

GAPDH

Figure 3B

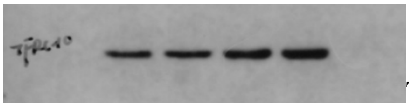

TFRC

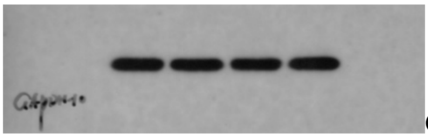

GAPDH

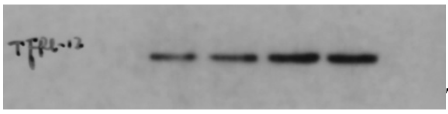

TFRC

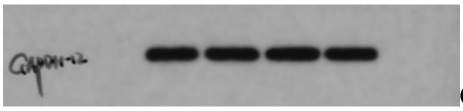

GAPDH

Figure 3C

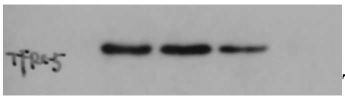

TFRC

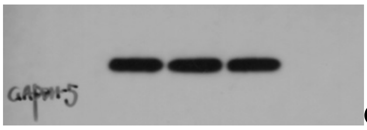

GAPDH

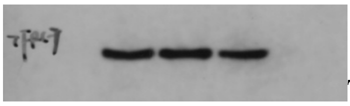

TFRC

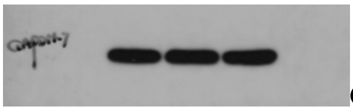
GAPDH

Figure 4A

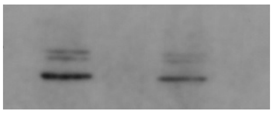
RAB17

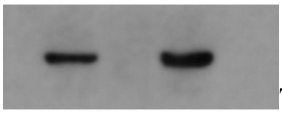
TFRC

Figure 4B

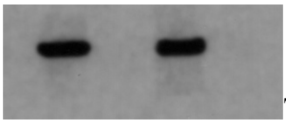
TFRC

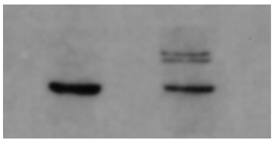
RAB17

Figure 4C

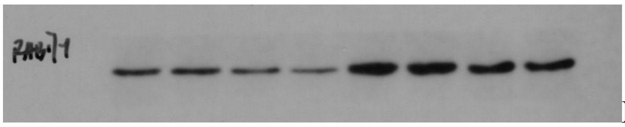
RAB17

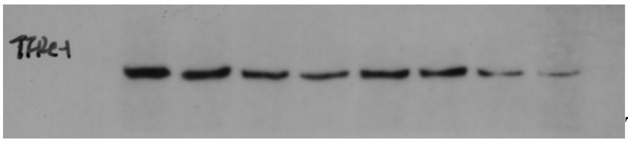
TFRC

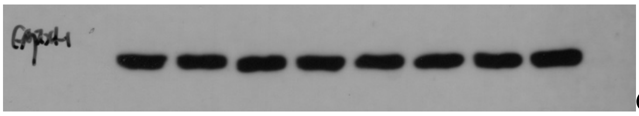
GAPDH

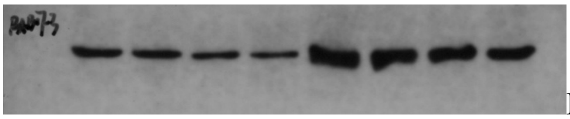
RAB17

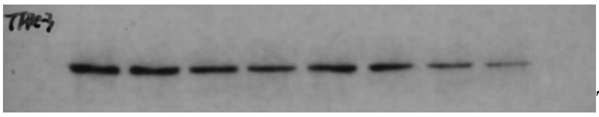
TFRC

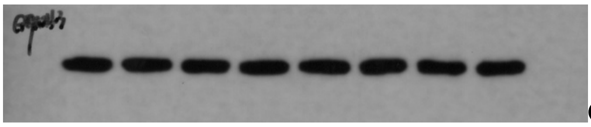
GAPDH

Figure 4D

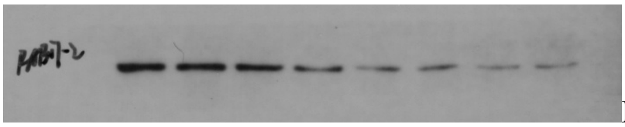
RAB17

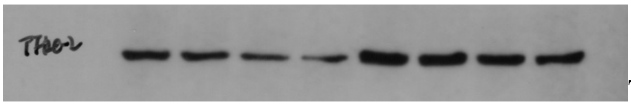
TFRC

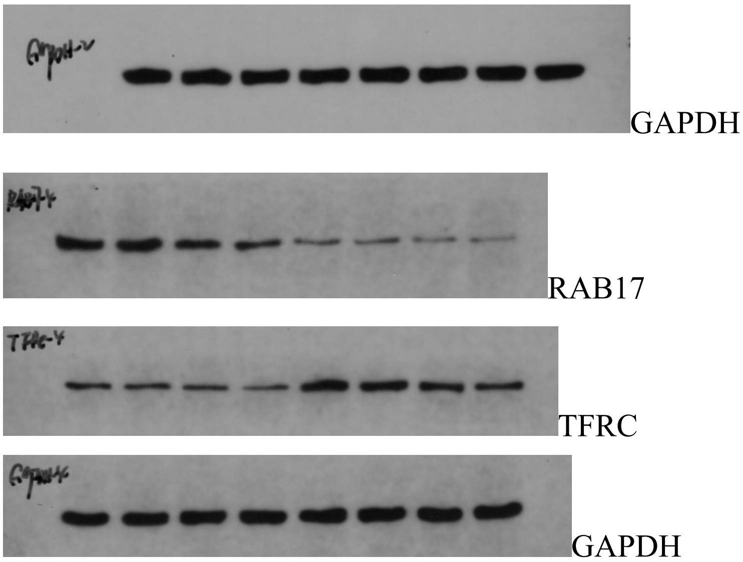

Figure 4E

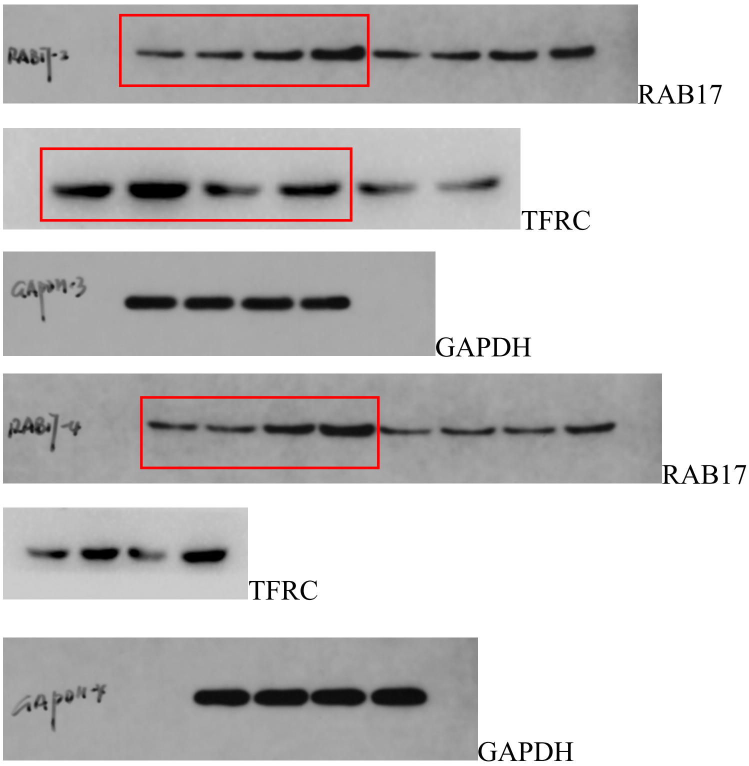

Figure 4F

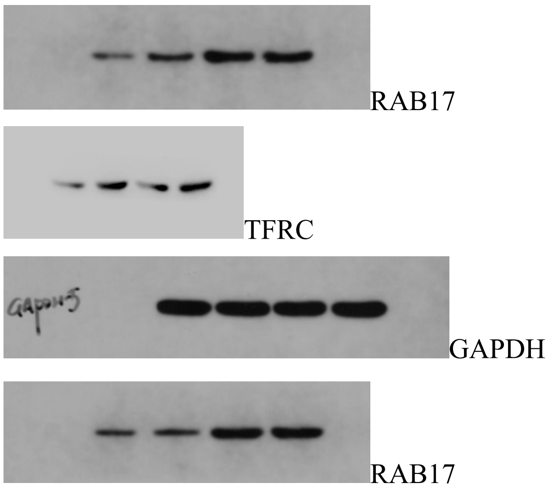

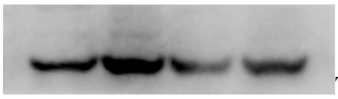
TFRC

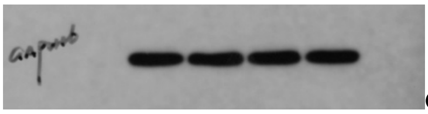
GAPDH

Figure 4G

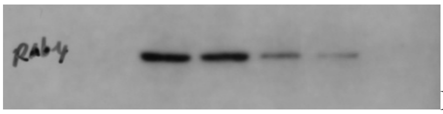
RAB17

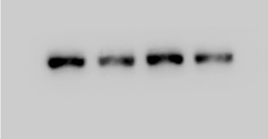
TFRC

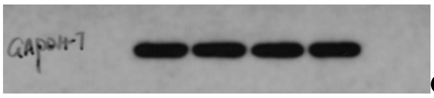
GAPDH

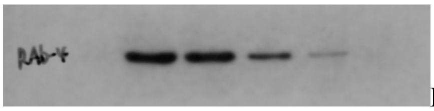
RAB17

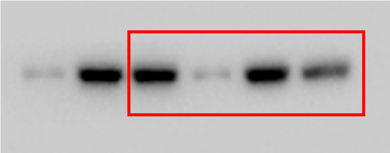
TFRC

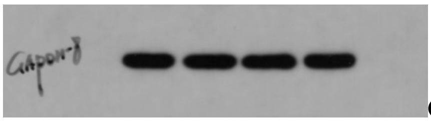
GAPDH

Figure 4H

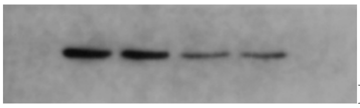
RAB17

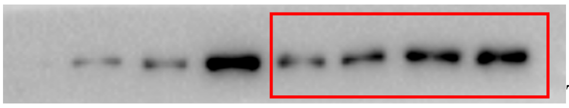
TFRC

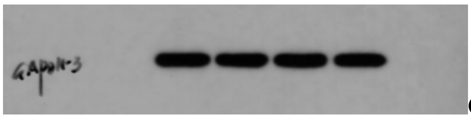
GAPDH

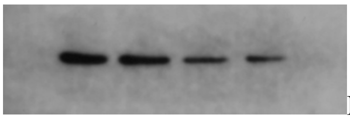
RAB17

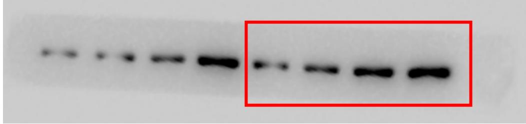
TFRC

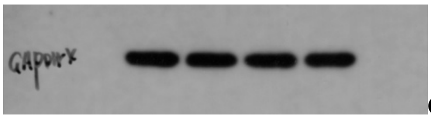
GAPDH

Figure 4I

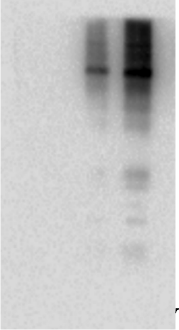

TFRC

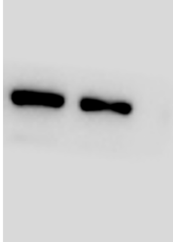

GAPDH

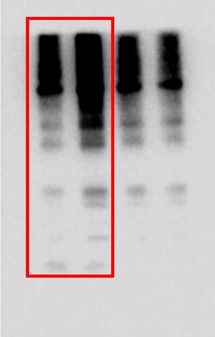

TFRC

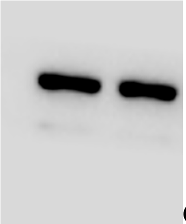

GAPDH

Figure 4J

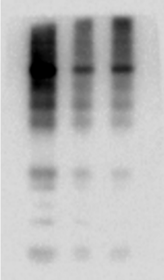

TFRC

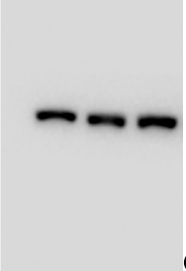

GAPDH

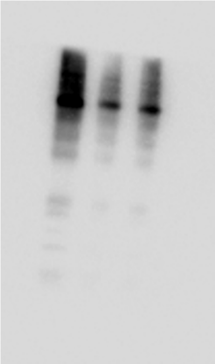
TFRC

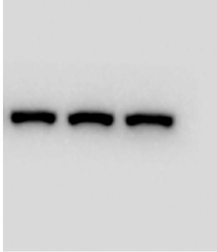
GAPDH

Figure 4K

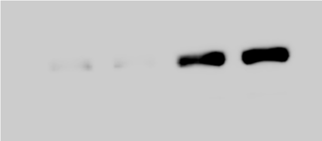
NEDD4L

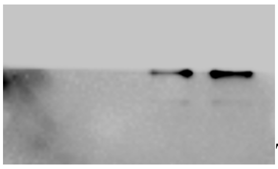
TFRC

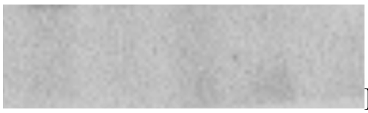
RAB17

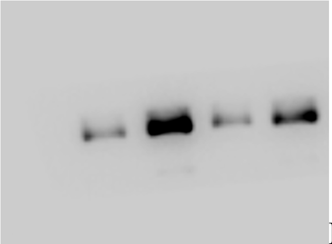
RAB17

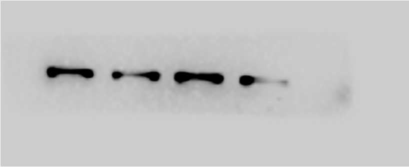
TFRC

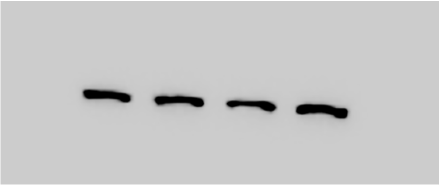
NEDD4L

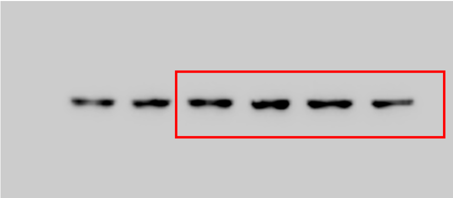

GAPDH

Figure 4L

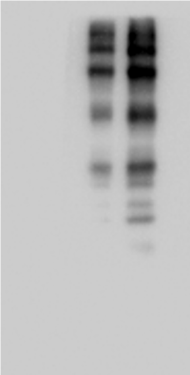

TFRC

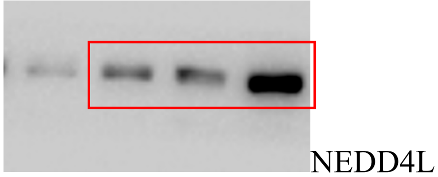

NEDD4L

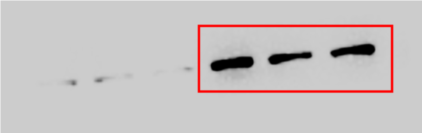

RAB17

Figure 4M

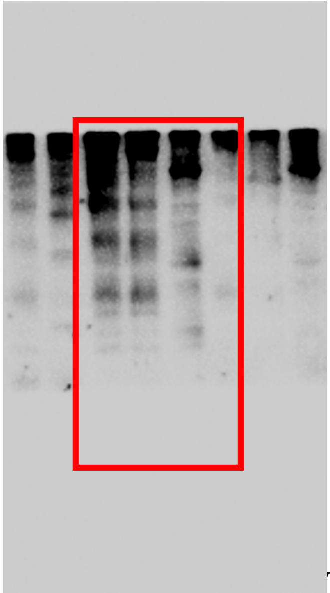

TFRC

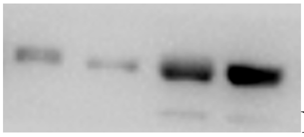

NEDD4L

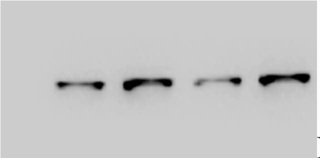
RAB17

Figure 5C

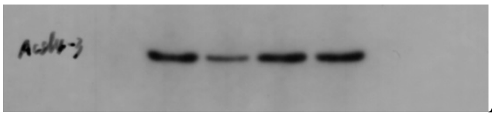
ACSL4

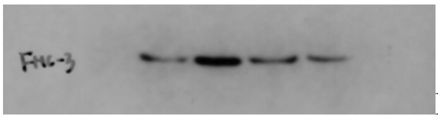
FHC

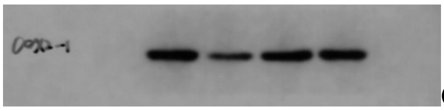
COX2

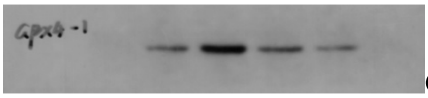
GPX4

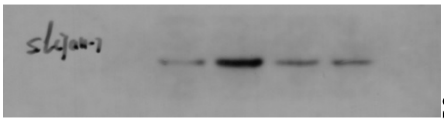
SLC7A11

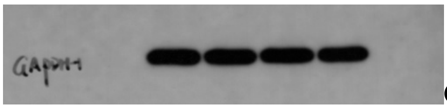
GAPDH

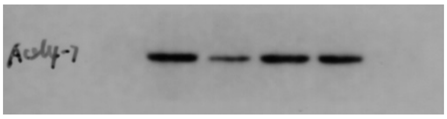
ACSL4

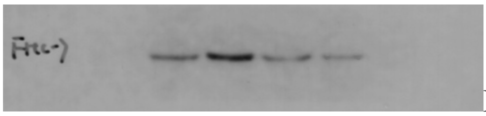
FHC

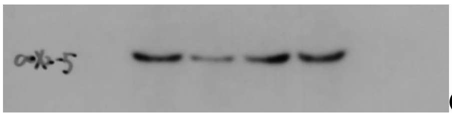
COX2

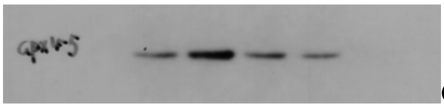
GPX4

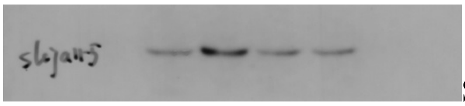
SLC7A11

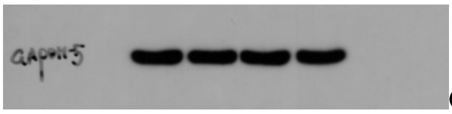
GAPDH

Figure 5D

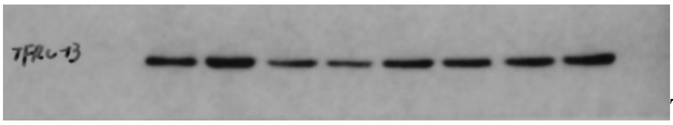
TFRC

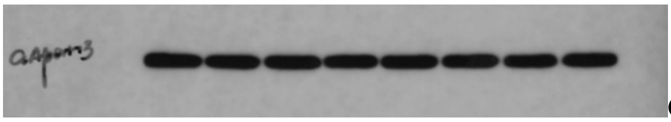
GAPDH

Figure 6A

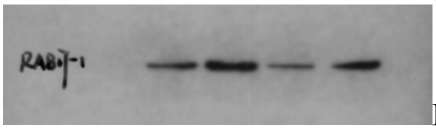
RAB17

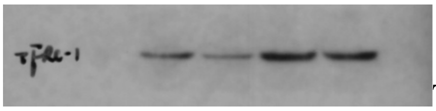
TFRC

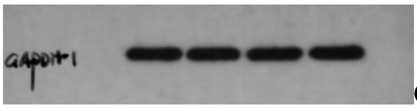
GAPDH

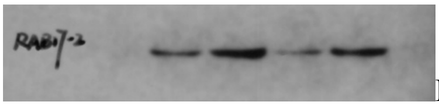
RAB17

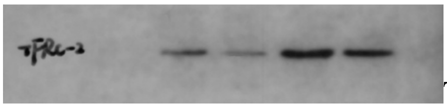
TFRC

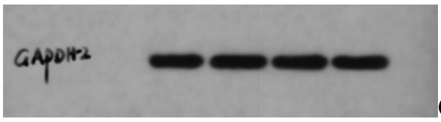
GAPDH

Figure 6E

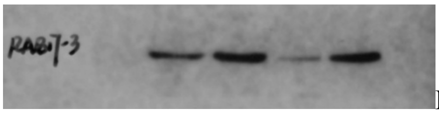
RAB17

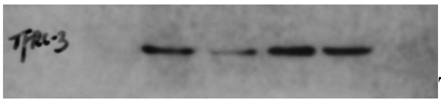
TFRC

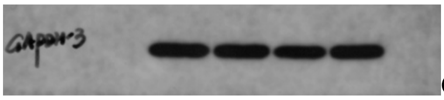
GAPDH

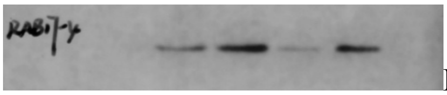
RAB17

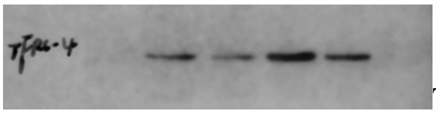
TFRC

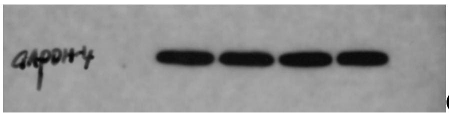
GAPDH

Figure 7D

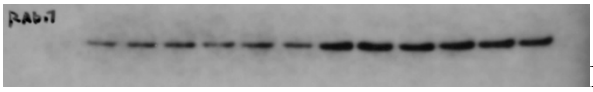
RAB17

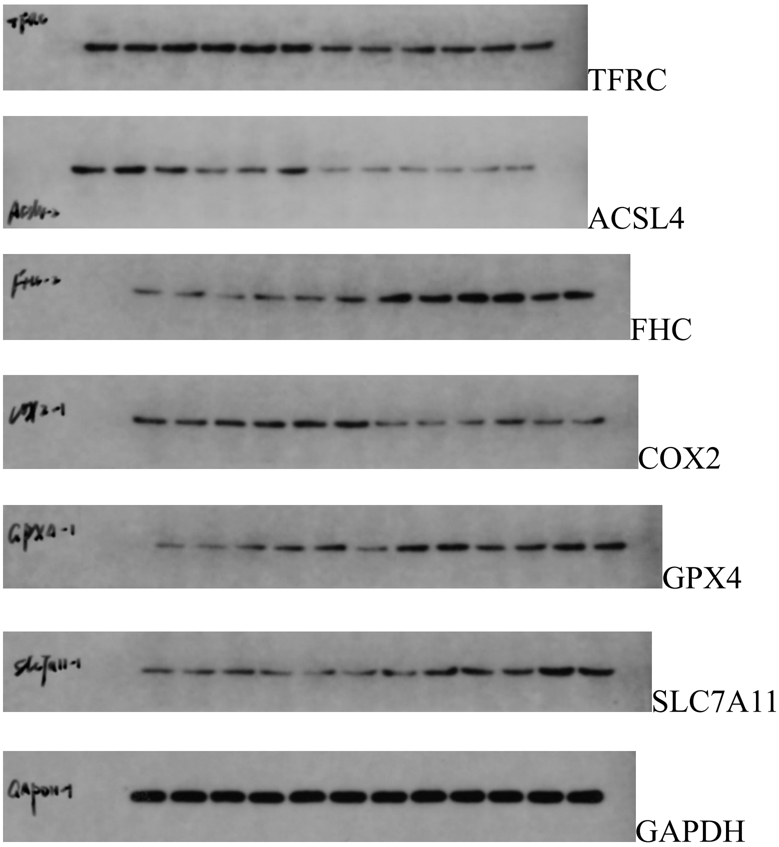

Figure S5D

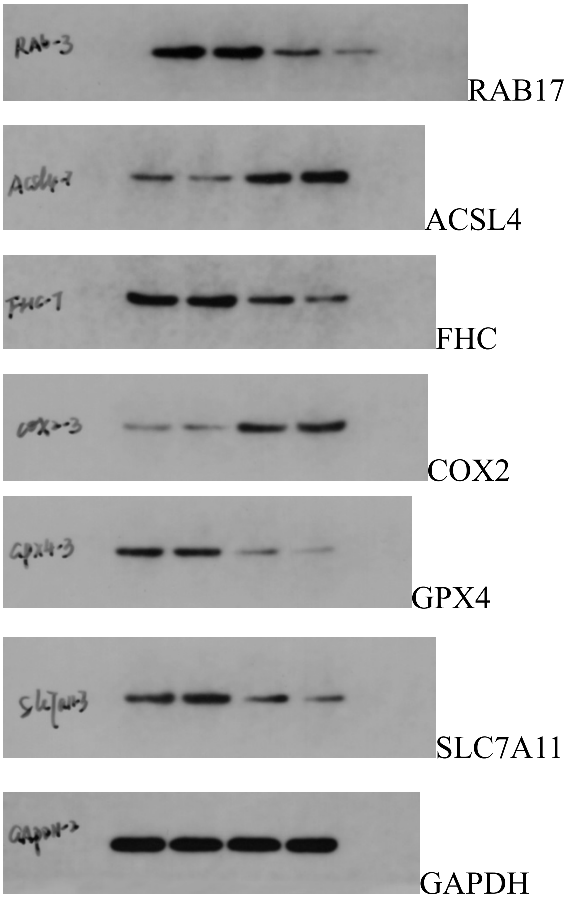

Figure S5E

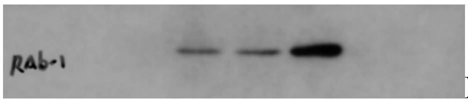
RAB17

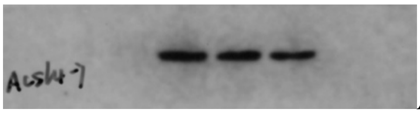
ACSL4

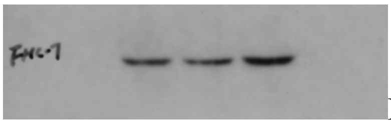
FHC

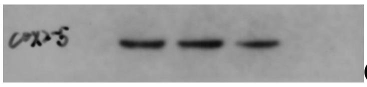
COX2

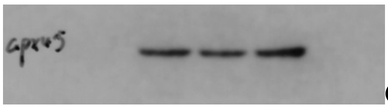
GPX4

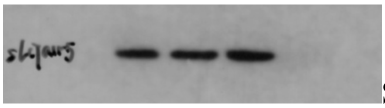
SLC7A11

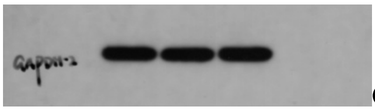
GAPDH
